# DNA-based identification of predators of the corallivorous Crown-of-Thorns Starfish (*Acanthaster* cf. *solaris*) from fish faeces and gut contents

**DOI:** 10.1038/s41598-020-65136-4

**Published:** 2020-05-18

**Authors:** Frederieke J. Kroon, Carine D. Lefèvre, Jason R. Doyle, Frances Patel, Grant Milton, Andrea Severati, Matt Kenway, Charlotte L. Johansson, Simon Schnebert, Peter Thomas-Hall, Mary C. Bonin, Darren S. Cameron, David A. Westcott

**Affiliations:** 1 Australian Institute of Marine Science, Townsville, Qld 4810 Australia; 2 Great Barrier Reef Marine Park Authority, Townsville, Qld 4810 Australia; 3 CSIRO Land and Water, Atherton, Qld 4883 Australia

**Keywords:** Tropical ecology, DNA sequencing, Marine biology

## Abstract

The corallivorous Crown-of-Thorns Starfish (CoTS, *Acanthaster* spp.) has been linked with the widespread loss of scleractinian coral cover on Indo-Pacific reefs during periodic population outbreaks. Here, we re-examine CoTS consumption by coral reef fish species by using new DNA technologies to detect Pacific Crown-of-Thorns Starfish (*Acanthaster* cf. *solaris*) in fish faecal and gut content samples. CoTS DNA was detected in samples from 18 different coral reef fish species collected on reefs at various stages of CoTS outbreaks in the Great Barrier Reef Marine Park, nine of which had not been previously reported to feed on CoTS. A comprehensive set of negative and positive control samples confirmed that our collection, processing and analysis procedures were robust, although food web transfer of CoTS DNA cannot be ruled out for some fish species. Our results, combined with the (i) presence of CoTS spines in some samples, (ii) reported predation on CoTS gametes, larvae and settled individuals, and (iii) known diet information for fish species examined, strongly indicate that direct fish predation on CoTS may well be more common than is currently appreciated. We provide recommendations for specific management approaches to enhance predation on CoTS by coral reef fishes, and to support the mitigation of CoTS outbreaks and reverse declines in hard coral cover.

## Introduction

Crown-of-Thorns Starfish (CoTS, *Acanthaster* spp.) are carnivorous starfish that feed on living tissue of scleractinian (i.e. hard) corals^1^. Across the Indo-Pacific, CoTS have been linked with the widespread loss of hard coral cover on reefs during periodic and recurrent population outbreaks^2^. For example, average hard coral cover halved on the Great Barrier Reef (GBR; Australia) from 1985 to 2012, with 42% of this decline attributed to coral predation by the Pacific Crown-of-Thorns Starfish (*Acanthaster* cf. *solaris*)^3^. Renewed outbreaks of CoTS are causing extensive coral loss on the GBR^4^, notably in areas that were mostly unaffected by recent mass bleaching events^5,6^. These high levels of CoTS predation on scleractinian corals have the potential to fundamentally alter the form and structure of coral reefs and their biological communities^1^.

Mitigating the detrimental impacts of CoTS predation, and reversing the declines in coral cover requires effective control and prevention of CoTS population outbreaks^3,7^. Current management interventions include manual control of individual starfish to reduce CoTS populations directly^6^, and reducing land-based run-off to lower recruitment rates of CoTS pelagic larvae into benthic settlement^8–10^. In addition, a variety of analyses have linked Marine Protected Areas (i.e. zoning) and CoTS outbreaks on the GBR, including (i) reefs closed to fishing having fewer CoTS than those open to fishing^11,12^, and (ii) the impacts of CoTS outbreaks being reduced at reefs closed to fishing^13^. These findings suggest an impact of zoning on CoTS population outbreaks^13^, with one potential pathway being a higher level of predation on one or more CoTS life stages on reefs closed to fishing^14^.

The release from predator pressure, i.e. the ‘*predator removal*’ hypothesis, has been posited as a potential contributor to CoTS population outbreaks^15^. Originally, a decrease in population densities of the Giant Triton (*Charonia tritonis*) was thought to underpin this release, being the only known predator of larger juvenile and adult CoTS at the time^15^. Since then, a total of 80 species of coral reef fishes and invertebrates have been identified as predators of planktonic and settled life stages of CoTS^16^. This would suggest that, rather than being influenced by just a single predator, the combined consumption of CoTS by a range of predators may mitigate the severity of CoTS population outbreaks^15,16^. Most of these records, however, comprise predation on injured and moribund or dead individuals under experimental conditions, with only a limited number of field observations of predation on early life stages and on healthy, un-injured adults^16^. Despite this lack of field observations of CoTS predation, several independent modelling studies have provided support for the ‘*predator removal*’ hypothesis^17–19^. Hence, a more comprehensive understanding of coral reef organisms that prey on live CoTS in the field, including on early life stages, is critical to inform active intervention in predation pressure to mitigate CoTS population outbreaks.

In this study, we re-examine CoTS consumption by coral reef fish species by using evidence of CoTS DNA detected in fish faecal and gut content samples. Specifically, we apply a new digital droplet PCR (ddPCR)-based method to detect DNA of the Pacific Crown-of-Thorns Starfish (*A.* cf. *solaris*)^20^ in samples from wild-caught fish. First, we conducted a literature review to target our field collections towards those coral reef fish species that are likely to consume the different life stages of CoTS, using Cowan *et al*.^16^ as a starting point. Second, we conducted two pilot studies to confirm our ability to detect CoTS DNA in fish faecal and gut content samples in both laboratory and field collected samples. Finally, we collected faecal and gut content samples from a total of 678 individual fish from 101 different coral reef fish species and 21 different families on reefs experiencing varying levels of CoTS outbreaks during and outside the CoTS spawning seasons^1,21^. Our results demonstrate that potential predation by coral reef fish on different life stages of CoTS is likely to be more widespread than is currently appreciated.

## Results

### Reports on Crown-of-Thorns Starfish predation by coral reef fish

To target our field collections towards those coral reef fish species that are likely to consume the different life stages of CoTS, a detailed literature review was conducted. A recent synthesis on known predators, reporting a total of 56 species of coral reef fish from 11 families feeding on CoTS (Cowan *et al*.^16^), was used as the starting point. Our review identified a total of 71 coral reef fish species from 16 families reported to have fed on *Acanthaster* spp. based on field and laboratory observations (Table 1). Fifty-two of these species fed on injured, moribund or dead CoTS with all but one feeding event observed under experimental conditions; 41 species were observed in field settings, 9 in both field and laboratory settings and 2 in laboratory settings. Most of these species (n = 42) have not been reported to consume living and un-injured CoTS. Field reports on predation on the early life stage of CoTS, including pelagic gametes and larvae and early post-settlement juveniles, are very limited^16^ at least in part due to the difficulties of observing such predation^1^. Indeed, such observations are restricted to three fish species, namely the Scissortail Sergeant (*Abudefduf sexfasciatus*) and Staghorn Damsel (*Amblyglyphidodon curacao*) consuming CoTS eggs^15,22^ and the Oriental Butterflyfish (*Chaetodon auripes*) eating CoTS sperm^23^ released during spawning. Recent laboratory feeding experiments, however, have reported an additional 11 damselfish feeding on spawned eggs and pelagic larvae, with several species readily taking to the food offered^24–26^. Field reports on predation on the settled life stage of CoTS are slightly more common^16^, having been reported for 12 fish species from the families Lethrinidae (emperor; 4 species), Balistidae (triggerfish; 2 species), Tetraodontidae (pufferfish; 2 species), Diodontidae (porcupinefish; 1 species), Labridae (wrasse; 1 species), Pomacanthidae (angelfish; 1 species) and Serranidae (grouper, 1 species) (Table 1). Remains of CoTS have been confirmed in gut contents of five of these fish species, namely Spotted Porcupinefish (*Diodon hystrix*)^27^, Humphead Maori Wrasse (*Cheilinus undulatus*)^27–29^, Redthroat Emperor (*Lethrinus miniatus*)^27^, Spangled Emperor (*Lethrinus nebulosus*)^30^ and Queensland Grouper (*Epinephelus lanceolatus*)^31,32^, and reported but not confirmed for Yellowmargin Triggerfish (*Pseudobalistes flavimarginatus*)^33^. Lethal predation by fish in the field on settled and apparent healthy CoTS has only been observed sporadically, namely by Stars-and-stripes Puffer (*Arothron hispidus*), Titan Triggerfish (*Balistoides viridescens*) and Spangled Emperor (*L. nebulosus*)^27^, with most studies reporting sublethal predation on CoTS arms^34,35^. Finally, *Acanthaster planci* DNA has been detected in gut contents using metabarcoding in a cardinal fish (*Nectamia savayensis*), Bigscale Soldierfish (*Myripristis berndti*) and Smallmouth Squirrelfish (*Sargocentron microstoma*)^36^.

**Table 1:** Predation on CoTS by coral reef fish reported in the literature. Review of studies in the primary and grey literature on the predation on different life stages of Crown-of-Thorns Starfish (CoTS, *Acanthaster* spp.) by coral reef fish species. (F) and (L) indicate field and laboratory-based observations, respectively.

**Species**	**Common name**	**CAAB number**	**Predation on different CoTS life stages**				**Location**	**References**
			**Pelagic**	**Benthic**	**Injured juvenile / adult**	**Moribund / dead juvenile / adult**		
Apogonidae								
*Nectamia savayensis*	a cardinalfish	37 327163	F^DNA^	F^DNA^			10	^36^
**Ballistidae**								
*Balistapus undulatus*	Orangestripe Triggerfish	37 465047				F	1	^41^
*Balistoides viridescens*	Titan Triggerfish	37 465048		F/L	L	L	1, 2, 3, 4	^27,29,41–43,82^
*Pseudobalistes flavimarginatus*	Yellowmargin Triggerfish	37 465071		F*/L			1, 5, 3, 4	^27,29,33,42^
*Rhinecanthus aculeatus*	Hawaiian Triggerfish	37 465028				L	1, 2	^43^
*Sufflamen verres*	Orangeside triggerfish	n/a			F	F	6	^80^
**Chaetodontidae**								
*Chaetodon aureofasciatus*	Goldstripe Butterflyfish	37 365013				F	1	^41,44^
*Chaetodon auriga*	Threadfin Butterflyfish	37 365019			L	F/L	1, 3	^41,42,44,81^
*Chaetodon auripes*	Oriental Butterflyfish	n/a	F				7	^23^
*Chaetodon baronessa*	Triangular Butterflyfish	37 365034				F	1	^44^
*Chaetodon citrinellus*	Citron Butterflyfish	37 365036			F	F	8	^44,80^
*Chaetodon kleinii*	Klein’s Butterflyfish	37 365040				F	1	^44^
*Chaetodon lineolatus*	Lined Butterflyfish	37 365041				F	1	^44^
*Chaetodon plebeius*	Bluespot Butterflyfish	37 365050				F	1	^41^
*Chaetodon rafflesi*	Lattice Butterflyfish	37 365052				F	1	^41^
*Chaetodon rainfordi*	Rainford’s Butterflyfish	37 365053				F	1	^41,44^
*Chaetodon ulietensis*	Doublesaddle Butterflyfish	37 365060				F	1	^44^
*Chaetodon vagabundus*	Vagabond Butterflyfish	37 365062				F/L	1, 2	^41,43,44^
**Diodontidae**								
*Diodon hystrix*	Spotted Porcupinefish	37 469015		F^G^			1	^27^
**Gobiidae**								
*Cryptocentrus* sp.	Shrimpgoby	n/a				F	1	^81^
**Holocentridae**								
*Myripristis berndti*	Bigscale Soldierfish	37 261006	F^DNA^	F^DNA^				^36^
*Sargocentron microstoma*	Smallmouth Squirrelfish	37 261027		F^DNA^				^36^
**Labridae**								
*Cheilinus fasciatus*	Redbreast Maori Wrasse	37 384066				F	1	^81^
*Cheilinus undulatus*	Humphead Maori Wrasse	37 384038		F^G^			1, 9	^27–29^
*Coris caudimacula*	Spot-tail Wrasse	37 384092				F	1	^41^
*Halichoeres melanurus*	Hoeven’s Wrasse	37 384032				F/L	1, 2	^43,44^
*Oxycheilinus digrammus*^1^	Violetline Maori Wrasse	37 384065				F	1	^81^
*Thalassoma hardwicki*	Sixbar Wrasse	37 384165				F	1	^81^
*Thalassoma jansenii*^2^	Jansen’s Wrasse	37 384166				F	1	^41^
*Thalassoma lucasanum*	Cortez Rainbow Wrasse	n/a			F	F	6	^80^
*Thalassoma lunare*	Moon Wrasse	37 384167				F/L	1, 2	^41,43,44^
**Lethrinidae**								
*Lethrinus atkinsoni*	Yellowtail Emperor	37 351013		F		F	1	^41,44,83^
*Lethrinus laticaudus*	Grass Emperor	37 386001				F	1	^44^
*Lethrinus miniatus*^3^	Redthroat Emperor	37 351009		F^G^		F	1	^27,44,83^
*Lethrinus nebulosus*	Spangled Emperor	37 351008		F^G^		F	1	^27,30,41,44,84^
*Monotaxis grandoculis*	Bigeye Seabream	37 351026		F^M^			1	^83^
**Lutjanidae**								
*Lutjanus bohar*	Red Bass	37 346029				F	1	^44^
*Lutjanus gibbus*	Paddletail	37 346028				F	1	^44^
**Mullidae**								
*Parupeneus multifasciatus*	Banded Goatfish	37 355026				F	1	^41^
**Nemipteridae**								
*Scolopsis bilineatus*	Two-line Monocle Bream	37 347031				F	1	^41,44^
**Pomacanthidae**								
*Holacanthus passer*	King Angelfish	n/a		F	F	F	6	^80^
*Pomacanthus semicirculatus*	Blue Angelfish	37 365080				F	1	^81^
*Pomacanthus sexstriatus*^4^	Sixband Angelfish	37 365010				F	1	^27,44,81^
**Pomacentridae**								
*Abudefduf sexfasciatus*	Scissortail Sergeant	37 372011	F/L				1	^15,24,26^
*Acanthochromis polyacanthus*	Spiny Puller	37 372015	L			F/L	1, 2	^24,26,43,44^
*Amblyglyphidodon curacao*^5^	Staghorn Damsel	37 372017	F/L			F	1	^22,24–26,44^
*Chromis atripectoralis*	Blackaxil Puller	37 372036	L				1	^24–26^
*Chromis margaritifer*^6^	Two-tone Chromis	37 372146	L				1	^85^
*Chromis viridis*^7^	Blue-green Puller	37 372053	L		L	F/L	1, 3	^24–26,42,81^
*Chrysiptera cyanea*	Blue Demoiselle	37 372060	L				1	^25^
*Chrysiptera rollandi*	Bluehead Demoiselle	37 372067	L				1	^24,25^
*Dascyllus aruanus*	Banded Humbug	37 372073	L				1	^24,25^
*Dascyllus reticulatus*	Headband Humbug	37 372074	L				1	^24–26^
*Dischistodus melanotus*	Blackvent Damsel	37 372077				F	1	^44^
*Neoglyphidodon melas*	Black damsel	37 372084				F	1	^41,44^
*Neoglyphidodon oxyodon*	Bluestreak Damsel	37 372137				F	1	^41^
*Neopomacentrus azysron*	Yellowtail Demoiselle	37 372087	L				1	^24,26^
*Pletroglyphidodon dickii*	Dick’s Damsel	n/a				F	1	^44^
*Pomacentrus amboinensis*	Ambon Damsel	37 372106	L				1	^24–26^
*Pomacentrus chrysurus*	Whitetail Damsel	37 372110				F	1	^41^
*Pomacentrus moluccensis*^8^	Lemon Damsel	37 372118	L		L	F/L	1, 3	^24–26,41,42,81^
*Pomacentrus wardi*	Ward’s Damsel	37 372127				F	1	^41^
*Stegastes acapulcoensis*^9^	Acapulco Major	n/a			F	F	6	^80^
*Stegastes nigricans*	Dusky Gregory	37 372135				F	1	^41^
**Scaridae**								
*Scarus ghobban*	Bluebarred Parrotfish	37 386001				F	1	^44^
**Serranidae**								
*Epinephelus lanceolatus*^10^	Queensland Grouper	37 311061		F^G^			n/a	^31,32^
**Tetraodontidae**								
*Arothron hispidus*	Stars-and-stripes Puffer	37 467033		F/L	F	F/L	1, 6, 3, 2, 4	^27,29,41–44,80,82,86^
*Arothron manilensis*	Narrowlined Puffer	37 467020			F	F/L	1, 3	^41,80^
*Arothron meleagris*	Whitespotted Pufferfish	37 467064			F	F	6	^80^
*Arothron nigropunctatus*	Blackspotted Puffer	37 467027				F	1	^81^
*Arothron stellatus*	Starry Puffer	37 467014		F			1	^23^

^DNA^ = CoTS DNA detected in gut contents, with potential CoTS life stage eaten inferred from known dietary items reported in FishBase (https://www.fishbase.se/); ^*^CoTS remains reported but not confirmed in gut contents; ^G^ = CoTS remains confirmed in gut contents; ^M^ = CoTS mouthed only. Numbers in superscript denote synonyms used in literature reviewed: ^1^ = *Cheilinus digrammus*; ^2^ = *Thalassoma nigrofasciatum*; ^3^ = *Lethrinus chrysostomus*; ^4^ = *Euxiphipops sexstriatus, E. sextinatus*; ^5^ = *Abudefduf curacao*; ^6^ = *Chromis dimidiata*; ^7^ = *Chromis caerulea*; ^8^ = *Pomacentrus popei*; ^9^ = *Eupomacentrus acapulcoensis*; ^10^ = *Promicrops lanceolatus*. Locations: 1 = Great Barrier Reef; 2 = Papua New Guinea; 3 = Philipines; 4 = Red Sea; 5 = Fiji; 6 = Panama; 7 = Okinawa; 8 = Guam; 9 = Marshall Islands; 10 = Moorea; n/a = not available.

### Can CoTS DNA be detected in fish faecal and gut content samples?

The ability to detect DNA from Pacific Crown-of-Thorns Starfish (CoTS, *A.* cf. *solaris*) in fish faecal and gut content samples was confirmed in two pilot studies (Supplementary Text 1). First, CoTS DNA (mtCOI gene fragment) was detected in faecal samples collected from the Blackspotted Puffer (*Arothron nigropunctatus*) fed freshly-killed CoTS in controlled laboratory settings, with detection up to seven days post-feeding in two of the five pufferfish (Supplementary Fig. 1.1). In addition, faecal samples preserved in 100% EtOH and liquid nitrogen showed similar amplification for CoTS mtCOI fragments (both 919 bp and 126 bp), providing confidence in the use of 100% EtOH as a preservative during field trips in remote locations (Supplementary Fig. 1.2). Second, faecal and gut content samples were collected from a range of coral reef fish species in the field, with CoTS spines detected in faeces from wild-caught Spangled Emperor (*L. nebulosus*) and Starry Puffer (*Arothron stellatus*) (Supplementary Fig. 1.3). Learnings from these two pilot studies were applied during subsequent field collections, in particular around the collection and preservation (Supplementary Text 2) and preventing contamination (Supplementary Text 3) of faecal and gut content samples when CoTS DNA is potentially present in the environment^20,21^.

### Collection of faeces and gut contents from coral reef fish species

**Figure 1:**
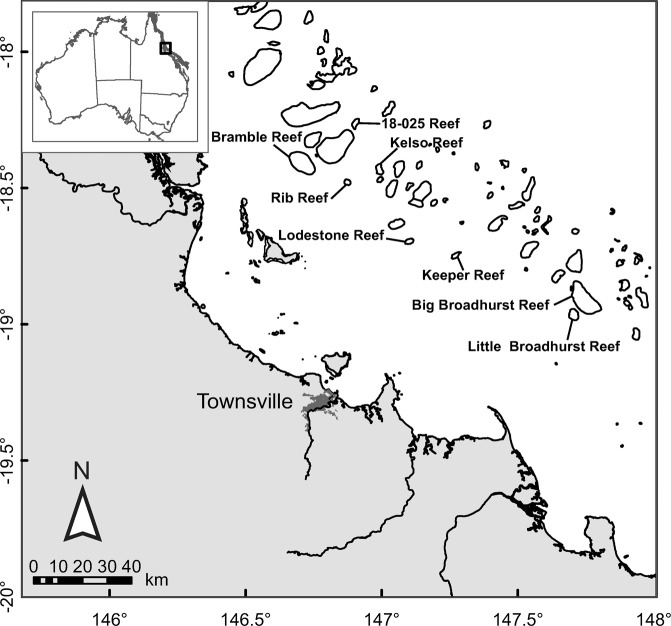
Sampling locations. Locations of coral reef fish collections at eight midshelf reefs in the central Great Barrier Reef World Heritage Area, Australia, conducted from the RV Cape Ferguson in 2018 and 2019. The status of CoTS population outbreaks varied from no outbreak to severe across the eight reefs at the time of fish collection (Table 2). Insert shows location of study area in Australia. The spatial layers to create the map were obtained from the Great Barrier Reef Marine Park Authority under a Creative Commons Attribution 4.0 licence (CC BY) (http://www.gbrmpa.gov.au/about-us/resources-and-publications/spatial-data-information-services).

A total of 678 individual fish from 101 different coral reef fish species and 21 different families were collected on reefs experiencing varying levels of CoTS population outbreaks in January 2018, July 2018 and July 2019 (Figure 1; Table 2; Supplementary Text 2; Supplementary Table 2.1). During the CoTS breeding season^1,21^ in January 2018, a total of 418 individuals from 59 different fish species were collected (range 1 to 21 individuals per fish species). Outside the CoTS breeding season^1,21^, in July 2018, a total of 173 individuals from 37 different fish species were collected (range 1 to 18 individuals per fish species). During both these trips, fish were kept overnight on the RV Cape Ferguson for collection of their faeces the next morning; faecal material was observed for most of these fish (for 405 and 138 individual fish, respectively). During the third field trip conducted in July 2019, speared fish were kept on ice and dissected on return to the RV Cape Ferguson for collection of their gut content. A total of 87 individuals from 19 different fish species were collected (range 1 to 10 individuals per fish species); gut content material was present in all fish.

**Table 2:** Sampling locations of coral reef fish.

**Reefs**			**CoTS outbreak status**^b^		
**Name**	**Number**	**Zone**^a^	**Jan-18**	**Jul-18**	**Jul-19**
Unnamed	18-025	Marine National park	Established	Potential	n/a
Bramble	18-029	Habitat Protection	Severe	Severe	n/a
Kelso	18-030	Marine National Park; Habitat Protection	No outbreak*	No outbreak*	Established
Rib	18-032	Habitat Protection	Severe	Severe	n/a
Lodestone	18-078	Habitat Protection	n/a	Severe	n/a
Keeper	18-079	Habitat Protection	n/a	n/a	Severe
Big Broadhurst	18-100a, b	Habitat Protection	n/a	n/a	No outbreak^#^
Little Broadhurst	18-106	Habitat Protection	n/a	n/a	Potential

Collection information for three field trips conducted on the Great Barrier Reef (GBR) in 2018 and 2019, from the AIMS Research Vessel (RV) Cape Ferguson (January and July 2018, July 2019). For each reef, zoning type ^69,70^ and Pacific Crown-of-Thorns Starfish (CoTS, *Acanthaster* cf. *solaris*) population outbreak status are given. n/a = not applicable; cells shaded in grey denote reefs visited during each of the three field trips.

^a^Activities allowed, prohibited or requiring a permit in the different zones are: *Habitat protection*: Open and fished; trawling prohibited, large mesh gill netting allowed; *Marine National Park*: No take; Extractive use prohibited without the GBRMPA’s permission^71^.

^b^CoTS population outbreak status information from GBRMPA’s Eye on the Reef, following definitions in De’ath (2003)^87^. ‘No outbreak’ status does not mean reefs are totally CoTS free. ^*^CoTS present at certain locations of Kelso Reef but overall below outbreak threshold status. ^#^Anecdotal accounts of CoTS presence at Big Broadhurst reef.

### Detection of CoTS DNA in faeces and gut contents from coral reef fish species

**Figure 2:**
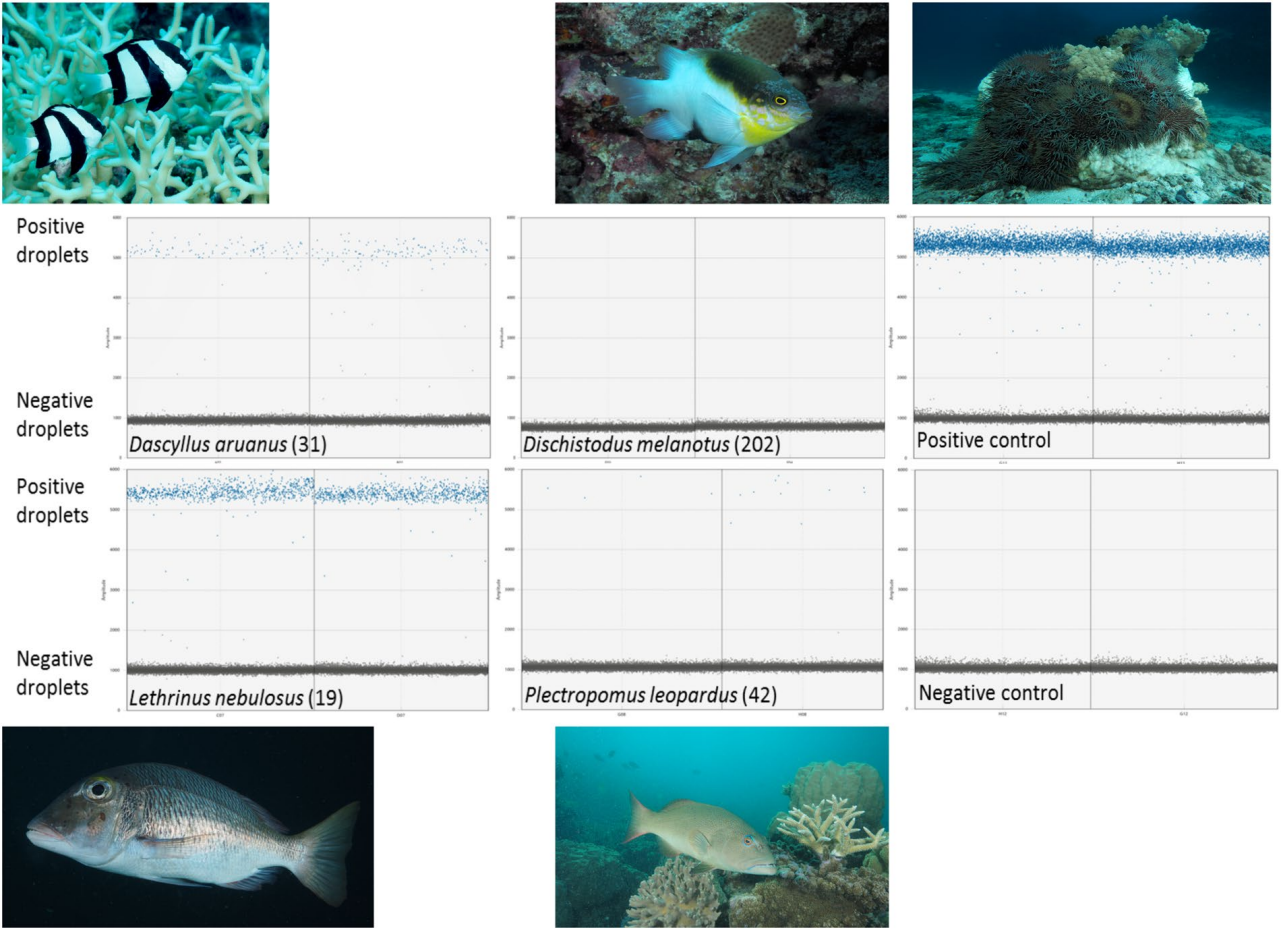
Detection of CoTS DNA in fish faecal and gut content samples. Examples for positive and negative digital droplet PCR results for four different coral reef fish, namely Banded Humbug (*Dascyllus aruanus*; positive), Blackvent Damsel (*Dischistodus melanotus*; negative), Spangled Emperor (*Lethrinus nebulosus*; positive), and Common Coral Trout (*Plectropomus leopardus*; positive). Sample collection number for each individual fish are given. Examples of digital droplet PCR results for positive (one to two 8-day old *Acanthaster* cf. *solaris* larvae) and negative (blanks) controls are also provided.

CoTS DNA was detected in faecal and gut content samples from a total of 30 individuals from 18 different coral reef fish species and eight different families (Table 3). During the CoTS spawning season (January 2018), CoTS DNA was detected in the faeces of seven individual fish from six different damselfish species (Table 3; Fig. 2). For each of these six species, this represented ≤50% of intraspecies samples collected at the reefs where positive CoTS DNA detections on faecal samples were made. One of these species, namely the Neon Damsel (*Pomacentrus coelestis*), has previously not been reported feeding on CoTS (Table 1). For all seven fish, faecal matter (albeit no distinct CoTS remains) was present in the holding bag or after filtration through the mesh sieve. The size range for these seven fish ranged from 20 to 52 mm Standard Length (SL), suggesting young of the year for all but two species namely the Banded Humbug (*Dascyllus aruanus*) and Neon Damsel (*P. coelestis*). All seven fish were collected from two reefs (Bramble, Rib) that experienced severe CoTS outbreak status at the time of collection (Table 2). CoTS DNA was detected in plankton samples collected at Bramble and Rib Reef indicating the presence of CoTS gametes and/or larvae (Supplementary Text 3; Supplementary Table 3.1)^21^. In contrast, no CoTS DNA was detected in plankton samples or faecal matter from fish collected at the two other reefs (Unnamed, Kelso), despite an established CoTS outbreak at Unnamed Reef and CoTS present at certain locations of Kelso Reef but overall being below outbreak threshold status (Table 2).

**Table 3:** Detection of CoTS DNA in fish faecal and gut content samples.

**Fish species**				**Positive detection**			
**Family**	**Species**	**Common name**	**CAAB number**	**N (totals)**	**SL (mm)**	**Reef (Outbreak status**^**a**^**)**	**Mon-Yr**
Pomacentridae	*Acanthochromis polyacanthus*	Spiny Puller	37 372015	1 (2; 17)	36	Rib (S)	Jan-18
	*Dascyllus aruanus*	Banded Humbug	37 372073	1 (6; 20)	40	Rib (S)	Jan-18
	*Neoglyphidodon melas*	Black Damsel	37 372084	1 (4; 4)	32	Bramble (S)	Jan-18
	*Pomacentrus amboinensis*	Ambon Damsel	37 372106	1 (3; 17)	20	Bramble (S)	Jan-18
	*Pomacentrus chrysurus*	Whitetail Damsel	37 372110	1 (3; 16)	31	Bramble (S)	Jan-18
	*Pomacentrus coelestis*^*#*^	Neon Damsel	37 372111	2 (5; 15)	44, 52	Rib (S)	Jan-18
Balistidae	*Balistapus undulatus*	Orangestripe Triggerfish	37 465047	1 (2; 2)	160	Rib (S)	Jul-18
Haemulidae^#^	*Diagramma pictum labiosum*^*#*^	Painted Sweetlips	37 350003	1 (2; 13)	330	Rib (S)	Jul-18
Labridae	*Cheilinus chlorourus*^*#*^	Floral Maori Wrasse	37 384064	1 (8; 9)	150	Lodestone (S)	Jul-18
Lethrinidae	*Gymnocranius grandoculis*^*#*^	Robinson’s Seabream	37 351005	1 (1; 1)	390	Kelso (N*)	Jul-18
	*Lethrinus lentjan*^*#*^	Redspot Emperor	37 351007	3 (11, 11)	215, 240, 250	Rib (S)	Jul-18
	*Lethrinus miniatus*	Redthroat Emperor	37 351009	2 (5, 2; 22)	290, 420	Rib (S), Lodestone (S)	Jul-18
	*Lethrinus nebulosus*	Spangled Emperor	37 351008	5 (9; 18)	320 (3×), 340, 390	Rib (S)	Jul-18
	*Lethrinus ornatus*^*#*^	Ornate Emperor	37 351015	3 (8; 12)	200, 250, 290	Rib (S)	Jul-18
Lutjanidae	*Lutjanus fulviflama*^*#*^	Blackspot Snapper	37 346034	2 (9; 12)	190 (2×)	Rib (S)	Jul-18
	*Lutjanus russelli*^*#*^	Moses’ Snapper	37 346065	1 (2; 6)	240	Lodestone (S)	Jul-18
Serranidae	*Epinephelus cyanopodus*^*#*^	Purple Rockcod	37 311145	1 (1; 1)	280	Kelso (N*)	Jul-18
Tetraodontidae	*Arothron nigropunctatus*	Blackspotted Puffer	37 467027	2 (4; 4)	110, 140	Unnamed (P)	Jul-18

^a^ = CoTS population outbreak status information from GBRMPA’s Eye on the Reef, following definitions in De’ath (2003)^87^; S = severe outbreak, N = No outbreak, P = Potential outbreak. ‘No outbreak’ status does not mean reefs are totally CoTS free. ^*^CoTS present at certain locations of Kelso Reef but overall below outbreak threshold status. ^*#*^ = Denotes coral reef fish species and families for which prior observations of feeding on COTS do not exist.

DNA from the Pacific Crown-of-Thorns Starfish (CoTS, *Acanthaster* cf. *solaris*) detected in faecal and gut content samples from coral reef fish species collected on mid-shelf reefs at various stages of CoTS outbreaks on the Great Barrier Reef Marine Park, Australia. CoTS DNA was detected in 30 individuals from 18 different coral reef fish species and eight different families. For each fish species, the number of individuals that tested positive for CoTS DNA, their size (Standard Length, SL) and their sample location and time are given. Totals in between brackets represent sample size at sample reef and time where positive detections were made, and sample size across all three sampling trips (January 2018, July 2018 and 2019).

Outside the CoTS spawning season (July 2018 and 2019), CoTS DNA was detected in faecal and gut content samples of 23 individual fish from 12 different fish species and seven families (Fig. 2; Table 3). For seven of these species, this represented ≥50% of intraspecies samples collected at the reefs where positive CoTS DNA detections on faecal and gut content samples were made. Eight of these species, namely the Painted Sweetlips (*Diagramma pictum labiosum*), Floral Maori Wrasse (*Cheilinus chlorourus*), Robinson’s Seabream (*Gymnocranius grandoculis*), Redspot Emperor (*Lethrinus lentjan*), Ornate Emperor (*Lethrinus ornatus*), Blackspot Snapper (*Lutjanus fulviflama*), Moses’ Snapper (*Lutjanus russelli*) and Purple Rockcod (*Epinephelus cyanopodus*) have previously not been reported feeding on CoTS (Table 1). For all but three fish, faecal matter (including distinct CoTS remains in three Spangled Emperor, *L. nebulosus*) was present in the holding crate or after filtration on the mesh sieves. The size range for these 23 fish ranged from 110 to 420 mm SL (Table 3), suggesting all individuals were 1^+^ year classes for all species. The 23 fish were collected from four reefs (Unnamed, Kelso, Rib, Lodestone) that experienced different levels of CoTS outbreaks (Table 2).

### Preventing contamination of faecal and gut content samples

To prevent and examine potential contamination of fish faecal and gut content samples from CoTS DNA present in the environment^20,21^, a comprehensive set of negative and positive control measures were taken prior and during field trips (Supplementary Text 3). For negative control measures, this included confirming the removal of CoTS DNA from unfiltered seawater following treatment in our filtration system on-board the RV Cape Ferguson in July 2018 (Supplementary Table 2.3; Supplementary Fig. 2.1). In addition, no CoTS DNA was detected in faecal samples collected from all but one fish species assigned as negative controls, namely from six different herbivorous species (n = 12 individuals) from four different families, and from six different corallivorous species (n = 15 individuals) from two different families (Supplementary Table 3.1). CoTS DNA was detected in one out of 17 samples collected from adult Common Coral Trout (*Plectropomus leopardus*), an obligate piscivore when an adult^37^, suggesting that food web transfer of CoTS DNA cannot be ruled out for some of the fish species that tested positive for CoTS DNA. Moreover, CoTS DNA was absent in all faecal samples collected from 13 different fish species (n = 97 individuals) on reefs at Lizard Island without CoTS population outbreaks during the CoTS spawning season in January 2019 (Supplementary Table 3.2). Individuals of these 13 fish species (of similar sizes as those collected at Lizard Island) including adult Common Coral Trout, collected on reefs with CoTS outbreaks on the central GBR in January and July 2018, had tested positive for CoTS DNA (Table 3; Supplementary Table 3.1). Equally important, our processing and preserving procedures did not destroy CoTS DNA as exemplified by the positive detection of CoTS DNA in water samples collected from the CoTS aquarium in the National Sea Simulator, and in 100% EtoH preserved freshly collected CoTS spines from the CoTS aquarium at the Lizard Island Research Station (Supplementary Table 3.1).

## Discussion

To inform active intervention in predation pressure to mitigate CoTS population outbreaks, a more comprehensive understanding of coral reef organisms that feed on live CoTS in the field, including on early life stages, is critical^16^. Here, we applied a ddPCR-based method to detect DNA of the Pacific Crown-of-Thorns Starfish (*A.* cf. *solaris*)^20^ in faecal and gut content samples from fish caught at reefs with varying levels of CoTS outbreaks. CoTS DNA was detected in samples from a total of 30 individuals from 18 different coral reef fish species and eight different families. Comparing these results with our literature review on CoTS predation by coral reef fish, nine of these fish species had not previously been reported feeding on CoTS. Our comprehensive set of negative and positive control measures were found to be robust, confirming that these positive detections were extremely unlikely to have resulted from contamination^38^ including with CoTS DNA present in the environment^20,21^. Food web transfer of CoTS DNA, however, cannot be ruled out for some of the fish species, based on one positive detection in adult Common Coral Trout (*P. leopardus*), an obligate piscivore species when adult^37^. Nonetheless based on known diet information for the coral reef fish species examined, the presence of CoTS spines in some samples, combined with reported lethal predation on CoTS gametes^15,22,23^ and larvae^24,26^ and lethal^27^ and sublethal^34,35^ predation on settled individuals, our results strongly indicate that direct fish predation on CoTS may well be more common than is currently appreciated.

Traditional gut content analysis has been largely ineffective in identifying putative CoTS predators and this method often fails to find remains of *Acanthaster* spp.^29,30,39^. Indeed, CoTS remains have only been confirmed in five coral reef fish species^27–32^, and reported but not confirmed for one other^33^. This is at least partly a result of fish being collected in areas with low^29^ or unknown^30^ abundance of adult *Acanthaster* spp., or of small sample sizes collected near areas with high CoTS densities^39^. In addition, identification of CoTS remains in the guts can be challenging and may easily be mistaken for other echinoderms^27,33,40^. Our application of a ddPCR-based method to detect *A.* cf. *solaris* DNA^20^ in faecal and gut content samples corroborated potential CoTS predation by known fish predators^16^ and identified previously unknown fish predators. Similarly, *A. planci* DNA has previously been detected in gut contents using metabarcoding in three other coral fish species^36^. These findings demonstrate that these methods can be applied to screen faecal or gut content samples of presumed CoTS predators, and validate field predation by fish and invertebrate species that have been reported to consume CoTS in experimental settings^41–44^. Importantly our study shows this can be achieved using non-invasive and non-lethal methods, a critical consideration for the ethical use of animals as well as for examining threatened species. Moreover, our ddPCR-based method detected CoTS DNA in a proportionally higher number of individuals in two emperor species (*L. miniatus*, *L. nebulosus*) compared to gut-content studies on the same species^27,30,39^. Similarly, CoTS remains were observed in only three of the 30 samples testing positive for CoTS DNA highlighting the superior sensitivity of this method versus gut content analyses. Increased detection of predation using qPCR versus visual gut content analysis has been reported for other fish species^45^. Hence, the ddPCR method would be a superior approach to identify additional CoTS predators on coral reefs across the Indo-Pacific^2^, and ascertain the frequency of CoTS consumption by known predators^16^.

Our findings strongly support the notion that coral reef fish feed on both the pelagic and benthic phases of CoTS in the field. Various behaviours by CoTS further corroborate that predation by visual predators is highly likely, including suggested spawning times during outgoing tides late afternoon and at night^23^, and highly cryptic and nocturnal behaviour by settled CoTS, particularly the smaller ones^46^. Consumption of the pelagic life stages of CoTS, i.e. gametes and larvae, has rarely been observed in the field^15,22,23^. Under controlled laboratory conditions, a wide range of damselfish species readily feed on CoTS eggs and larvae, including in the presence and in preference of Blue Seastar (*Linckia laevigata*) larvae^24,26^. This includes three of the six damselfish species that tested positive for CoTS DNA in our study. The confirmed presence of CoTS gametes and/or larvae at reef locations where the seven positive damselfish were collected, in combination with reported zooplankton diets for at least five of these six damselfish species^47^, indicate that feeding on pelagic CoTS larvae in the field is probable. Furthermore, the absence of CoTS DNA in faecal samples from obligate corallivorous and herbivorous fish species corroborates that these damselfish detections were not a result of feeding on coral mucus or filamentous algae contaminated with CoTS DNA. The relatively low incidence of CoTS DNA detection within individual damselfish species may well be related to a sporadic spatio-temporal distribution of the pelagic CoTS phase within a complex reef matrix^21,48^. Nevertheless, planktivorous fishes could still markedly reduce the number of CoTS larvae under such conditions, based on estimated CoTS consumption rates for damselfish in laboratory settings^24,26^ and high damselfish abundances in the field^49^.

Predation on the benthic phase of CoTS, i.e. juveniles and adults, has been observed slightly more often in the field^16^, with reports for at least eleven fish species. Our results strongly suggest that predation by coral reef fish on settled CoTS is more common, based on (i) detection of CoTS DNA in samples from nine wild-caught fish species previously not reported feeding on CoTS; (ii) detection of CoTS remains in wild-caught Spangled Emperor (*L. nebulosus*) and Starry Puffer (*A. stellatus*); and (iii) laboratory observations on Blackspotted Puffer (*A. nigropunctatus*) (previously not reported feeding on live CoTS) readily consuming freshly-killed CoTS, and biting off arms from an apparent healthy juvenile CoTS (Kroon, unpublished). Benthic CoTS were present, including at outbreak levels, at all reef locations where these 23 positive fish were collected during the non-breeding season. For all 12 species identified, reported diet information indicate that predation on benthic CoTS in the field is likely. However, the presence of CoTS DNA in a sample from one adult *P. leopardus*, used as a negative control in our study given that adults are obligate piscivores^37^, reveals that food web transfer of CoTS remains or CoTS DNA cannot be ruled out for at least some fish species. Similarly, the presence of CoTS DNA in field-collected samples cannot distinguish between consumption of a live, moribund or dead benthic CoTS, although inferences from known dietary habits and field observations can inform this. Combining this information and our ddPCR results for fish species in the emperor (Lethrinidae) and tropical snapper (Lutjanidae) families, particularly for Spangled and Redthroat Emperor (*L. nebulosus* and *L. miniatus*), strongly indicates that they may well be critical predators on the benthic phase of CoTS.

Many of the coral reef fish species that have been reported to feed on *Acanthaster* sp, including those testing positive for CoTS DNA in our study, are subject to various fisheries across the Indo-Pacific^14^. In the GBR Marine Park, the commercial Marine Aquarium Fish Fishery targets over 600 different coral reef fish species^50^, including damselfish, pufferfish and triggerfish^51^. This commercial fishery dates back to the 1970s^52^ and collected between 130 000 and 260 000 coral reef fish annually between 1998 and 2008^50^. The Coral Reef Fin Fish Fishery, comprising commercial, recreational (including charters) and Indigenous fishers, targets a range of fish species that are known or likely CoTS predators including emperors (Lethrinidae) and tropical snappers (Lutjanidae)^53^. Species in these two families have been fished commercially and recreationally on the GBR back to at least the 1950s^54^. Currently, Redthroat Emperor (*L. miniatus*) is one of the two primary target species in this fishery, with commercial harvests ranging from 137 tonnes to 271 tonnes per year comprising 10% to 17% of the commercial component of this fisheries’ total harvest from 2009/10 to 2017/18^55^. Spangled Emperor (*L. nebulosus*) is the other emperor of fisheries’ significance, with commercial harvests averaging 56 tonnes per year in the fisheries^56^. The harvest estimates for the charter, recreational and indigenous fisheries for these two species are less reliable but of a similar order of magnitude^57,58^. Whether the individual or combined take of these various fisheries, and associated release from predator pressure^15^, has been playing a role in influencing CoTS population outbreaks remains unclear. Fishing does reduce densities of planktivorous damselfish and ‘secondary target’ species, principally lethrinids and lutjanids, being lower on offshore reefs open to fishing than those closed to fishing^59^. This strongly supports the notion that a higher level of predation on one or more CoTS life stages on reefs closed to fishing may underpin the reported impact of zoning on CoTS population outbreaks^13^. Hence, further examination of potential spatio-temporal relationship(s) between coral reef fish presence and abundance, coral reef fish harvest by various fisheries and CoTS population outbreaks on Indo-Pacific coral reefs, including the GBR Marine Park, is warranted.

Potential mitigation of CoTS population outbreaks by coral reef fish is most likely to occur through lethal predation on the pelagic phase^15,22–26^ regulating the settlement of CoTS larvae, and lethal and sub-lethal predation on the settled phase influencing growth, reproduction^35^ and mortality^27^, in particular during low, non-outbreak, densities^1^. In the context of the GBR Marine Park, this would suggest that enhanced and targeted management of coral reef fish to mitigate CoTS outbreaks would be most effective in the initiation zone, i.e. the midshelf reefs between Lizard Island and Cairns^1,15,60–62^ during pre-outbreak conditions. The progressive southward spread of these outbreaks could also potentially be mitigated by management of coral reef fish, in particular on reefs that are identified as key nodes in COTS outbreak and spread processes^63,64^. Such management approaches may involve (i) reduced fisheries take of single or a suite of coral reef fish species known to consume CoTS; (ii) augmentative strategies to increase the abundance of single or a suite of coral reef fish species known to consume CoTS; (iii) temporal closures of reefs to fishing when environmental conditions conducive to outbreaks are predicted; and/or (iv) establishing new marine reserves (i.e. no-take areas) in the outbreak initiation zone and on highly connected reefs further south. These approaches would enhance fisheries management reforms currently being implemented, including delivery of sustainable fisheries catch limits to ensure 60% of the biomass remains to deliver more resilient marine ecosystems^65^. They would also complement current management interventions such as direct manual control^6^ and improving water quality in land-based run-off^8–10^. Given that CoTS population outbreaks are a major driver of the reported and projected decline in hard coral cover on the GBR^3,7^, current fisheries management reforms need to be fully implemented to contribute positively to reversing this decline. These additional proposed management approaches to mitigate CoTS outbreaks by enhancing coral reef fish predation should be seriously considered to improve hard coral cover now and into the future, both on the GBR and on Indo-Pacific reefs more broadly.

## Methods

### Study area

The GBR extends for 2,000 km along Australia’s north-eastern coast (Fig. 1) and contains a variety of tropical marine ecosystems including ~20 000 km^2^ of coral reefs, ~43 000 km^2^ of seagrass meadows and extensive mangrove forests (Great Barrier Reef Marine Park Authority, 2014). The 344,400 km^2^ GBR Marine Park was established under the Federal Great Barrier Reef Marine Park Act 1975^66^, followed with the listing of the 348,000 km^2^ GBR World Heritage Area (WHA) by UNESCO in 1981^67^. Since 1975, the GBR has been managed as a multiple-use area by the Australian Federal and Queensland State Governments^68^. The main commercial and non-commercial uses are commercial marine tourism, defence activities, fishing, ports, recreation (not including fishing), research and educational activities, shipping, and traditional use of marine resources^68^. Since 2004, these multiple uses have been managed under the Zoning Plan for the GBR Marine Park outlining activities allowed, prohibited or requiring a permit in the seven different zones^69,70^. The three major zone types are ‘General Use’ (open and fished; 33.8% of the GBR Marine Park), ‘Habitat Protection’ (open and fished except for trawling; 28.2%), and ‘Marine National Park’ (no-take; 33.3%)^71^.

### Crown-of-Thorns Starfish outbreaks on the GBR

The first and second CoTS population outbreaks on the GBR were noticed at Green Island and nearby reefs in 1962^15,60^ and in 1979^61^, respectively (Fig. 1). The first stages of the third CoTS population outbreak were detected in 1993, with outbreaks recorded on midshelf reefs between Lizard Island and Cairns in 1994-95^62^ (Fig. 1). A fourth outbreak is now in progress in the central and southern GBR^72^ which again was first detected on midshelf reefs between Lizard Island and Cairns in 2010^1^.

The four recorded CoTS population outbreaks on the central GBR seem to have followed a similar pattern of initiation and spread^1^. The first detection (or at least reporting) of high CoTS densities on midshelf reefs in the north-central section of the GBR suggests that outbreaks initiate roughly between Lizard Island (14.6°S) and Cairns (17°S)^15,60–62,73^ (the ‘initiation box’). The limited spatial and temporal resolution of reef monitoring in this area precludes the identification of single or multiple reefs as the source of primary outbreaks^73,74^. Following initiation, each of the four outbreaks shows a progressive southward spread at a rate of 1° of latitude every three years^1,75–77^. This is consistent with southward dispersal of CoTS larvae spawned at the outbreak front^75^. After approximately 15 years, CoTS population outbreaks appear to die off on midshelf reefs in the Pompeys’ section (21.0°–22.0° S) of the southern GBR^1^. Northward spread of CoTS population outbreaks from the initiation zone has also been reported^77^, although the pattern of spread is less clear due to fewer surveys having been conducted in the northern GBR.

### Life history of Crown-of-Thorns Starfish

The life history of *Acanthaster* spp. consists of a relatively short planktonic stage (i.e. days to weeks) and a longer settled stage (i.e. years) (all information from^1^, and references therein, unless otherwise noted). The planktonic stage begins with the release of female and male gametes which can include up to 65 million eggs per individual female. On the GBR, spawning generally occurs at the start of the summer wet season from November to February^21^. Following fertilization the larval phase, ranging in size from 0.5 to 1.5 mm long, can last from 9 to 42 days depending on temperature and food availability. Larvae settle onto reef habitat, showing a strong association with crustose coralline algae, and subsequently metamorphosize from a planktonic larva into a benthic juvenile seastar (0.5 mm diameter) over a period of two days. For the next six months, the juvenile seastar (1–10 mm diameter) will feed on crustose coralline algae before a permanent shift in diet to polyps of reef-building corals. The coral-feeding juvenile and sub-adult stages (10–200 mm diameter) last approximately two years, after which they sexually mature into a coral-feeding adult stage (200–350 mm diameter) which lasts two to five years. After five years, senile adult CoTS (>350 mm in diameter) generally show a decline and subsequent cessation of gametogenesis. It is likely that at all stages of their life history CoTS are exposed to predation by a variety of coral reef organisms, including by coral reef fishes^16^.

### Collection of faeces and gut contents from coral reef fish species

Field and laboratory work on fish described in this study was conducted in accordance with relevant guidelines and regulations, under permits from the Great Barrier Reef Marine Park Authority, the Queensland Department of Agriculture and Fisheries and the James Cook University Animal Ethics Committee.

To examine fish faecal and gut content samples for CoTS DNA, coral reef fish were collected at midshelf reefs experiencing varying levels of CoTS population outbreaks during three field trips in 2018 and 2019 (Table 2). To target our field collections towards those coral reef fish species that are likely to consume the different life stages of CoTS, we first conducted a literature review using Cowan *et al*.^16^ as a starting point. Specific information was sourced from the primary sources, including on (i) fish species (common and scientific name; family; Codes for Australian Aquatic Biota (CAAB) number), (ii) status of CoTS being consumed (pelagic or benthic stage; injured, moribund or dead), (ii) whether predation was observed in the field or laboratory, and (iv) location of predation.

In parallel with this literature review, we conducted two pilot studies to examine the feasibility to detect DNA from the Pacific Crown-of-Thorns Starfish (CoTS; *A.* cf. *solaris*) in fish faecal samples. The first pilot study examined whether CoTS DNA could be detected in fish faecal samples collected from dog faced pufferfish (*A. nigropunctatus*) fed freshly-killed CoTS in controlled laboratory settings (Supplementary Text 1; Supplementary Figs. 1.1; 1.2). The second pilot study examined whether faecal samples, potentially containing CoTS DNA, could be collected using non-lethal and non-invasive methods from coral reef fish in the field (Supplementary Text 1; Supplementary Fig. 1.3).

Based on the findings of the review and two pilot studies, a list of fish species was developed to target for collection during three field trips. To further refine our collections towards those fish species likely to consume different life stages of CoTS, we collated and reviewed information on diet and food items for each of these species from FishBase^78^ and associated primary sources, and from several reports by the Food and Agriculture Organization of the United Nations on fish and fisheries species in the Indo-Pacific region (Supplementary Table 2.2). For some species no information on diet, food items or primary sources was presented on Fishbase; in these cases we also searched the Web of Science. Based on dietary information we included certain fish species as ‘negative controls’, i.e. species that were highly unlikely to consume CoTS such as obligate herbivores, corallivores and piscivores (Supplementary Text 3; Supplementary Table 2.2). For all fish species, detailed procedures were implemented for the collection and preservation of their faecal and gut content samples (Supplementary Text 2). The first field trip, conducted during the CoTS spawning season^1,21^ in January 2018, targeted fish species that may consume early life history stages of CoTS (gametes, planktonic larvae and newly settled juveniles) (Supplementary Text 2). The second and third field trip, conducted outside the CoTS spawning season^1,21^ in July 2018 and July 2019, targeted fish species that may consume settled life history stages of CoTS (juveniles, sub-adults and adults) (Supplementary Text 2). The third and final field trip targeted specific coral reef fish species that proved difficult or impossible to capture using non-lethal methods during the July 2018 trip.

Based on the findings of the two pilot studies, a comprehensive set of negative and positive control measures were taken prior to and during these field trips (Supplementary Text 3), to prevent and examine potential contamination of fish faecal and gut content samples^38^ including with CoTS DNA present in the environment^20,21^. One key measure involved minimising the probability of false positives associated with fish predation on moribound and dead CoTS resulting from the CoTS control program run by the Great Barrier Reef Marine Park Authority (GBRMPA)^79^. All field collection trips on the central GBR were conducted on reefs that were experiencing CoTS outbreaks (Table 2), with some of these reefs being visited by the CoTS control program. This control program kills individual CoTS by using a single small volume injection of oxbile^41^; the dead CoTS are left on the reef to decompose. Four days after injection, little evidence of dead CoTS remain except for small piles of spines and skeletal elements^41^. Similar disintegration rates have been reported for moribund and dead CoTS with few spines and ossicles remaining after 4 to 8 days^80,81^. Fish predation on these moribund and dead CoTS could greatly increase the probability of detecting CoTS DNA in fish faeces and gut contents when no predation on life CoTS had occurred (i.e. false positive). Further, results from a preliminary study suggests that CoTS DNA decays exponentially in seawater to being undetectable after 8 days (Doyle, unpublished). To minimise the probability of false positives, we closely consulted with GBRMPA and only visited reefs that had either not experienced CoTS culling at all, or had not experienced CoTS culling for at least four weeks prior to the respective field trip. For the latter reefs, this would have allowed ample time for complete disintegration of culled CoTS (4–8 days) and complete decay of associated CoTS DNA in surrounding waters (8 days). For the January 2018 field trip, CoTS had never been culled (18-025) or had not been culled in the five weeks prior (Bramble, Kelso and Rib reefs). Similarly, for the July 2018 field trip, the final CoTS culling on Bramble, Rib, Kelso, Lodestone and 18-025 reefs took place at least five weeks prior (i.e. before or on 29 May 2018) and ceased until after this trip was finished in consultation with GBRMPA. Finally, for the July 2019 field trip, CoTS had not been culled (Kelso, Little and Big Broadhurst reefs) or had not been culled in the four weeks prior (Keeper).

### Processing faecal and gut content samples for detection of CoTS DNA

Fish faecal and gut content samples were preserved in 100% EtOH in sample vials ranging from 5 ml to 500 ml (Supplementary Text 2). Subsequent sample homogenisation, extraction and ddPCR, including assessing the ddPCR outcomes, were conducted by investigators who were unaware of the sample allocation (i.e. fish species). Preserved samples were homogenised in different ways depending on the sample volume. Faecal and gut content samples in 5 ml or 50 ml vials were homogenised in a bead-beater with stainless steel beads (BioSpec) for two minutes. For faecal samples captured over a filter disc (Supplementary Text 2), the disc was opened carefully into a cylindrical shape inside their sample vial and beads placed into the centre before being homogenised in a bead-beater (BioSpec) for two min. Gut content samples in 120 ml or 500 ml vials were homogenised using a commercial ‘paint shaker’ following the addition of stainless steel beads (BioSpec) for five min.

DNA extraction was conducted on a sub-sample of each homogenised sample. These sub-samples were taken after inverting the homogenised sample several times to ensure thorough mixing. For samples preserved and homogenised in 5 ml vials, a sub-sample of 1 ml was transferred to a 2 ml screw top tube. Following removal of EtOH using a rotary vacuum concentrator (Savant), DNA was extracted using slightly modified versions of the Qiagen DNeasy Blood and Tissue extraction kit. Briefly, a lysis solution of Qiagen buffer ATL (360 μl) and proteinase K (10 mg ml^-1^, 40 μl) was added followed by an overnight incubation (56 °C) with constant rotation. From each sample, 200 µl was then transferred into a new 2 ml microtube, which was placed in the sample rack within a Qiacube robot for automated DNA extraction using the following protocol. After addition of Qiagen buffer AL (200 µl), the ATL/proteinase K/AL solution incubated (56 °C) with agitation for 30 min. Ethanol (200 µl) was added to and mixed with the incubated solution. The ATL/proteinase K/AL/EtOH solution (600 µl) was loaded on to a Qiagen spin column and centrifuged at 10,000 × g for 1 min. Qiagen buffer AW1 (500 µl) was applied to the spin column followed by centrifugation at 10,000 × g for 1 min. Qiagen buffer AW2 (500 µl) was applied to the spin column followed by centrifugation at 20,000 × g for 3 min. Elution of DNA from the spin column was performed in 3 ×50 µl TE_(0.1)_. After each addition of 50 µl TE_(0.1)_, the spin column was incubated at room temperature for 1 min followed by a centrifugation at 10,000 g for 1 min.

For samples preserved and homogenised in 50 ml, 120 ml or 500 ml vials, the amount of sub-sample taken for DNA extraction was based on the original amount of faecal or gut content preserved. Specifically, these samples were grouped into three relative biomass categories namely ‘small’, ‘medium’ and ‘large’, and a sub-sample of 16 ml (small), 8 ml (medium) and 4 ml (large) was transferred into new 50 ml sample vials. Following removal of EtOH using a rotary vacuum concentrator (Savant), DNA was extracted as described above with the following modifications. The initial lysis solution added to the sub-sample consisted of Qiagen buffer ATL (1.8 ml) and proteinase K (0.2 ml, 10 mg ml^-1^). From each sample, 600 µl was transferred into a new 2 ml microtube, and the DNA extraction process completed on the Qiacube robot with a 600 µl volume used where a 200 µl was described above. In addition, the entire ATL/proteinase K/AL/EtOH solution (1800 µl) was loaded on to a Qiagen spin column in 3 ×600 µl batches. All other steps remained the same.

Digital droplet PCR on the smaller 126 bp CoTS mtCOI fragment, including positive and negative extraction controls as well as positive and negative PCR controls, was conducted following methods described in^20^. Blank extractions consisted of 5 ml or 50 ml tubes filled with 100% EtOH, and 1 ml or 4 ml sub-samples, respectively, processed as described above. Positive controls consisted of one to two 8-day old CoTS larvae added to 1 ml and 4 ml sub-samples and processed as a sample. Extraction controls (total: positive, n = 21; negative, n = 33) and PCR controls (total: positive, n = 38; negative, n = 40) were run for each batch of extractions.

To prevent potential contamination of fish faecal and gut content samples during laboratory processing and analyses^38^, the following measures were implemented. All pre- and post-PCR activities were kept physically separate by having a dedicated PCR room and equipment. For each batch of extractions, work-space and equipment used for sample processing such as pipettes were DNA cleaned (LookOut DNA Erase, Sigma). Implements used for sample processing such as tweezers and forceps were exposed to 100% bleach for at least 30 min, washed in Milli Q and dried in 100% EtOH prior to subsequent use on individual samples.

## Supplementary information

Supplementary information.

Supplementary information.

## Data Availability

All data needed to evaluate the conclusions in the paper are present in the paper and/or the Supplementary Information. Additional data related to this paper may be requested from the authors.
