# Author Correction: DNA-based identification of predators of the corallivorous Crown-of-Thorns Starfish (*Acanthaster* cf. *solaris*) from fish faeces and gut contents

**DOI:** 10.1038/s41598-020-75953-2

**Published:** 2020-10-28

**Authors:** Frederieke J. Kroon, Carine D. Lefèvre, Jason R. Doyle, Frances Patel, Grant Milton, Andrea Severati, Matt Kenway, Charlotte L. Johansson, Simon Schnebert, Peter Thomas-Hall, Mary C. Bonin, Darren S. Cameron, David A. Westcott

**Affiliations:** 1 Australian Institute of Marine Science, Townsville, Qld 4810 Australia; 2 Great Barrier Reef Marine Park Authority, Townsville, Qld 4810 Australia; 3 CSIRO Land and Water, Atherton, Qld 4883 Australia

Correction to: *Scientific Reports* 10.1038/s41598-020-65136-4, published online 18 May 2020

This Article contains an error in Figure 2, whereby the wrong species of fish is shown in the bottom left hand corner (image 4). The correct Figure 2 appears below as Figure 1.

**Figure 1:**
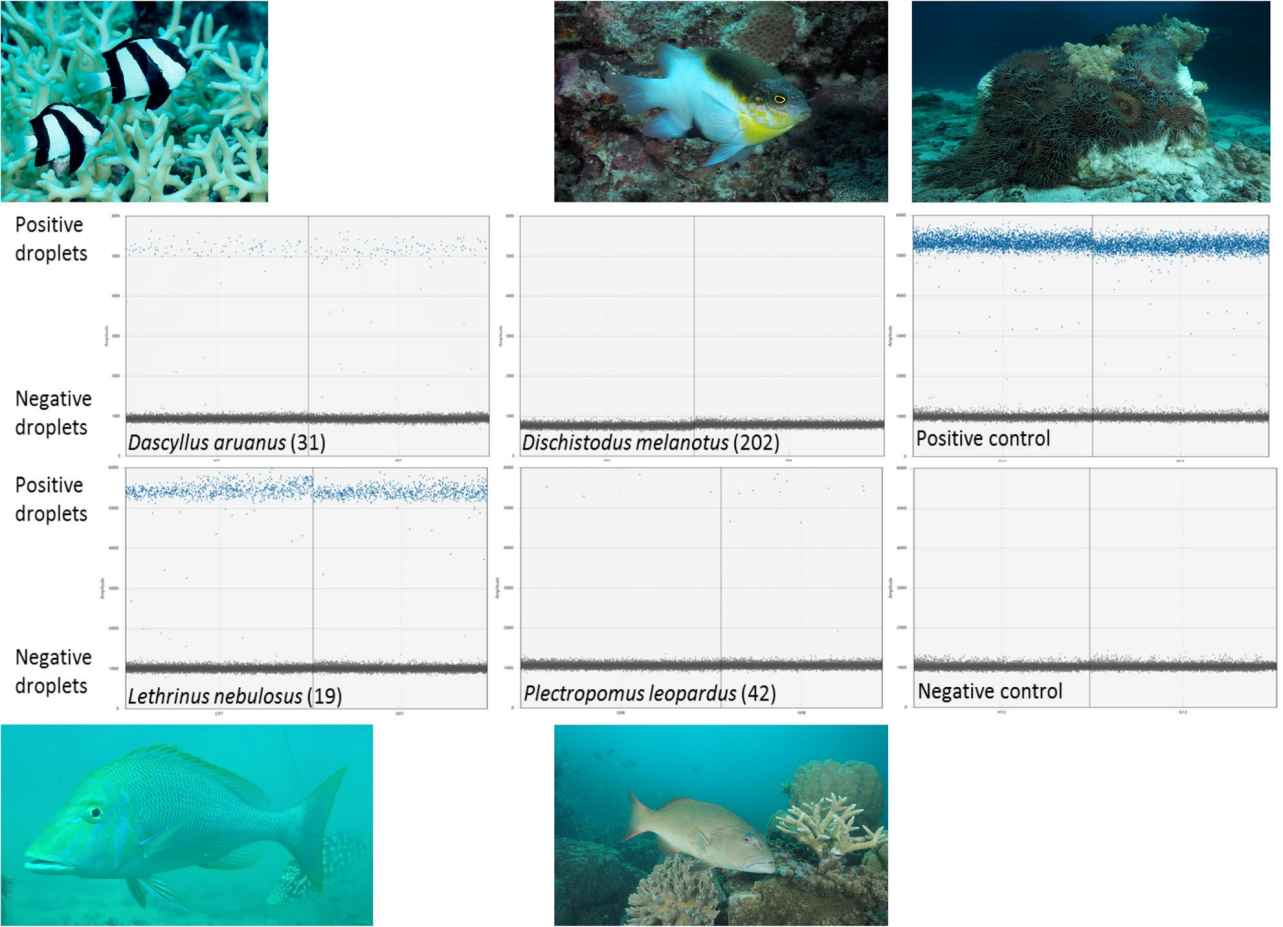
Detection of CoTS DNA in fish faecal and gut content samples. Examples for positive and negative digital droplet PCR results for four different coral reef fish, namely Banded Humbug (*Dascyllus aruanus*; positive), Blackvent Damsel (*Dischistodus melanotus*; negative), Spangled Emperor (*Lethrinus nebulosus*; positive), and Common Coral Trout (*Plectropomus leopardus*; positive). Sample collection number for each individual fish are given. Examples of digital droplet PCR results for positive (one to two 8-day old *Acanthaster* cf. *solaris* larvae) and negative (blanks) controls are also provided.

