# Supplementary material for: DNA-based identification of predators of the corallivorous Crown-of-Thorns Starfish (Acanthaster cf. solaris) from fish faeces and gut contents
Source: Sci Rep. 2020 May 18;10:8184. doi: 10.1038/s41598-020-65136-4 (PMC7235266; doi:10.1038/s41598-020-65136-4)
Supplement: Supplementary file 2 — Supplementary information. [file 41598_2020_65136_MOESM2_ESM.docx]

**DNA-based identification of predators of the corallivorous Crown-of-Thorns Starfish (*Acanthaster* cf. *solaris*) from fish faeces and gut contents**

**Frederieke J. Kroon^a,*^, Carine D. Lefèvre^a^, Jason R. Doyle^a^, Frances Patel^a^, Grant Milton^a^, Andrea Severati^a^, Matt Kenway^a^, Charlotte L. Johansson^a^, Simon Schnebert^a,^ Peter Thomas-Hall^a^,** **Mary C. Bonin**^b^**, Darren S. Cameron**^b^**, David A. Westcott^c^**

^a^ Australian Institute of Marine Science, Townsville, Qld 4810, Australia

^b^ Great Barrier Reef Marine Park Authority, Townsville, Qld 4810, Australia

^c^ CSIRO Land and Water, Atherton, Qld 4883, Australia

* Corresponding author: [f.kroon@aims.gov.au](mailto:f.kroon@aims.gov.au); tel.: +61-(0)7-4753-4159; ORCID ID 0000-0001-8771-6543

Short title: Reef fish predation on Crown-of-Thorns Starfish

# Supplementary Information

# Text 1. Laboratory and field pilot studies

In parallel with the literature review, and prior to embarking on the three main field trips, two pilot studies were conducted to examine the feasibility to detect DNA from Pacific Crown-of-Thorns Starfish (CoTS, *Acanthaster* cf. *solaris*) in fish faecal and gut content samples. Learnings from these two pilot studies were applied to the three main field trips, in particular around the collection and preservation of faecal and gut content samples and preventing contamination of such samples with CoTS DNA potentially present in the environment ^1,2^. Both pilot studies were conducted with permission from the Great Barrier Reef Marine Park Authority (GBRMPA), the Queensland Department of Agriculture and Fisheries (Qld DAF), and the James Cook University Animal Ethics Committee (JCU AEC).

## Pilot Study 1 – Can we detect CoTS DNA in fish faeces? - a laboratory experiment

This first pilot study examined whether DNA from the Pacific Crown-of-Thorns Starfish (CoTS; *Acanthaster* cf. *solaris*) could be detected in fish faecal samples. Specifically, we analysed faecal samples collected from Blackspotted Puffer (*Arothron nigropunctatus*) fed freshly-killed CoTS in controlled laboratory settings. Six Puffers were housed individually in 450 L circular holding tank with PVC tubes for shelter and with flow-through filtered seawater (nominal 0.2 µm) at the National Sea Simulator in Townsville, Australia. Holding tanks were kept inside at ambient temperature and light cycles. Fish were acclimatised for one week and fed squid *ad lib* for 20 min twice daily; fish that had not taken to the feed after seven days were not used in the pilot study (n = 1). The timing of this pilot study (August) was outside the CoTS spawning season (December to March) ^4^ to ensure that seawater would be free of CoTS larvae.

After acclimation, the five Puffers were given a one-off feed of small pieces of a freshly-killed, adult CoTS (time zero). Following the feeding on CoTS remains, each Puffer was gently transferred from their circular holding tank to a separate 60 L conical holding tank (400 mm diameter x 500 mm height) in the afternoon. The conical holding tanks were filled with filtered seawater (nominal 0.2 µm) and supplied with continuous airflow, and were also kept inside at ambient temperature and light cycles. The following morning each Puffer was returned to their individual holding tanks, and feeding of squid *ad lib* for 20 min resumed twice daily as per acclimation period. Faeces were collected from each individual conical holding tank by draining the contents through the outflow at the tapered bottom over a 40 μm mesh filter. Faeces caught on the mesh filter was transferred into sample preservation vials and stored in liquid nitrogen until further analysis for CoTS DNA. This process for collection of faecal samples was repeated with each individual fish every day for a total of seven days. Mesh filters and collection containers were cleaned with 10% bleach for at least 30 min and rinsed with copious amount of filtered seawater (nominal 0.2 µm) between individual collections.

To determine the suitability of three different preservation methods for fish faeces containing CoTS DNA, including for use during field trips in remote locations, we utilised sub-samples of faeces collected from four Puffers. Faeces collected on 40 µm mesh filters were distributed evenly among 2 ml sample vials, and preserved in either liquid nitrogen, 100% dimethylsulfoxide (DMSO), or 100% ethanol (EtOH) with the solvent preservation methods kept at room temperature.

All faecal samples collected during this pilot study were extracted using a Qiagen DNeasy kit according to the manufactures guidelines with the following exceptions: samples were incubated overnight (56°C) with shaking in Qiagen buffer ATL/Proteinase K (360 µl/40 µl respectively); incubation in Qiagen buffer AL (400 µl) was for 30 min; and the final elution was in 2 x 50 μl 10 mM Tris pH 8.0. PCR was conducted in 20 μl volumes using 2 μl of template DNA, 400 nM forward and reverse primers and AmpliTaq Gold 360 master mix. Two primers sets were utilised which are specific to the CoTS mitochondrial cytochrome oxidase subunit 1 gene (CoTS mtCOI) ^5,6^. One primer set amplifies a larger CoTS mtCOI fragment (919 bp) with the second primer set amplifying a smaller CoTS mtCOI fragment (126 bp). The primers amplifying the larger CoTS mtCOI fragment were COTS-COI-F-69 and COTS-COI-R-987 with the sequence GGCCTGAGCAGGAATGGTTGGAA and GCCTTGTAGCGTTGCCATTCACC, respectively ^6^. The second set of primers amplifying the small CoTS mtCOI fragment were denoted COTS-COI-F-1321 and COTS-COI-R-1446 and have the sequence TCCGACTACCCGGACGCCTATAC and AGTGGTTCGCTGGGAAGTGAAGG, respectively ^1^. After a 10 min activation step at 95°C, amplification followed a three-step profile of denaturation (95°C, 30 sec), annealing (60°C, 30 sec) and extension (72°C; 1 min 30 sec for 919 bp fragment, 30 sec for 126 bp fragment) for 35 cycles, with a final extension step at 72°C for 10 min. PCR amplification reactions were visualised on an agarose gel.

This pilot study confirmed that CoTS DNA can be detected in fish faecal samples. Specifically, CoTS DNA (mtCOI gene fragment) was detected in faecal samples collected from all five Blackspotted Puffers, with detection up to seven days post-feeding of freshly-killed CoTS in two of the five Puffers (Fig. 2.1). Successful amplification of targeted CoTS mtCOI fragments (both 919 bp and 126 bp) was achieved in most of the fish faeces samples utilised for the sample preservation trial (Fig. 1.2), indicating that detection of CoTS DNA in fish faecal samples is possible. Moreover, amplification of the smaller CoTS mtCOI fragment had a greater success for these samples compared to the large CoTS fragment (Fig. 1.2). This is most likely due to the digestion of CoTS DNA whilst passing through the gut passage of the fish ^7^. Samples preserved in 100% EtOH and liquid nitrogen showed similar amplification for CoTS mtCOI fragments (both 919 bp and 126 bp). One faecal sample (Fish 3) showed amplification of the 919 bp CoTS mtCOI fragment after preservation in 100% EtoH, but not in liquid nitrogen. In contrast, preservation of fish faecal samples in DMSO did not result in the same amplification success as preservation in either 100% EtOH or liquid nitrogen. Consequently, 100% EtOH was chosen at the preservation solvent of choice for future field-based collections.

## Pilot Study 2 – Refining collection methods for fish faecal samples – a field study

This pilot study examined whether faecal samples, potentially containing DNA from the Pacific Crown-of-Thorns Starfish (CoTS; *Acanthaster* cf. *solaris*), could be collected from coral reef fish in the field. Specifically, we aimed to refine collection methods for fish faecal samples using non-lethal and non-invasive methods. A range of coral reef fish species were collected on four midshelf reefs experiencing CoTS population outbreaks from the RV Cape Ferguson from 16 to 25 May 2017. The timing of this pilot study was also outside the CoTS spawning season (December to March) ^4^ to ensure that seawater used for keeping fish overnight would be free of CoTS larvae. Fish were collected on SCUBA at 1 to 9 m depth using either fence nets and/or hand nets and diluted clove oil solution ^8,9^ following JCU AEC Standard Operating Procedures (SoP numbers AQU-008 and AQU-012), or from the main vessel using a baited hook and fishing line. Following capture, fish were transported back to the vessel in holding buckets (22 L) or plastic crate (68 L) filled with aerated seawater and covered with lids.

On the vessel, individual fish were captured from holding buckets or plastic crates using a hand net, and transferred to individual plastic bags (small fish), 68 L plastic crates (medium fish), or 450 L circular holding tanks (large fish). Bags, plastic crates and holding tanks were filled with seawater mechanically filtered through two pressurised bag filters (Waterco C50) with 25 µm and 1 µm nominal mesh sizes, respectively. Bags containing one small fish each were closed with rubber banks and kept overnight in a styrofoam box placed in a 450 L circular holding tank and covered with a lid. Plastic crates and holding tanks containing one medium or large fish were aerated continuously overnight and also covered with a lid. All buckets, plastic crates, holding tanks, lids, air stones and air hoses were decontaminated with 10% bleach for at least 30 min and rinsed with filtered seawater prior to subsequent use. Plastic bags were only used once for faeces collection.

The next morning fish were removed from their bag, plastic crate or holding tank using a hand net for each fish, measured (Standard Length, SL, in mm) and either returned to the reef directly (for fish collected from the main vessel) or placed into one of the holding tanks with flow-through ambient seawater and covered with a lid for subsequent return to the site of collection. The contents of individual bags, plastic crates or holding tanks were filtered over a 40 µm mesh sieve, and the presence or absence of faecal material, including potential CoTS material, was noted for each fish. Gut contents were removed from those fish that had not survived overnight holding conditions and that had not defecated. Material in the mesh sieve was concentrated by either direct or indirect transfer into 5 ml or 50 ml preservation vial depending on the amount of material. For direct transfer, filtered seawater was used to concentrate the sample to an ‘edge’ of the mesh sieve and transferred into a 5 ml sample vial using 100% EtOH. The vial was then topped up with 100% EtOH. For indirect transfer, filtered seawater was used to transfer the sample onto a 47 mm Ø filter disc cut from a 40 µm nylon mesh placed on a filter holder within a filter funnel clamped to the set-up. The sample was subsequently passed through this filter disc under vacuum to simultaneously capture the faeces and remove seawater. The filter disc was removed from the filter holder and folded with the sample side on the inside using forceps, and transferred into a 5 ml preservation vial topped up with 100% EtOH. Where required, metal spatulas or forceps were used to concentrate and/or transfer material from mesh sieves. Prior to use, all mesh sieves, filter holders, filter funnels, clamps, spatulas, and forceps were decontaminated with 10% bleach for at least 30 min and rinsed with reverse osmosis (RO) water.

Measurements of seawater temperature (T, in ⁰C) and DO (in mg l^-1^) were taken in the morning from randomly selected bags, plastic crates or holding tanks. This enabled confirmation of maintaining quality of seawater in which individual fish were held overnight. Water quality was measured on all holding vessels in which fish had not survived overnight.

A total of 162 individual coral reef fish from 43 different species and eleven different families were collected as part of this field pilot study. Following overnight keeping of fish in bags, plastic crates, or holding tanks, faecal samples were present in almost all holding vessels. Echinoderm spines were observed in faeces from three individual fish from two species namely Spangled Emperor (*Lethrinus nebulosus*) (n = 2) and Starry Puffer (*Arothron stellatus*) (n = 1). Spines were visually checked for one individual from each fish species and confirmed to be from *Acanthaster* spp. ^3^ (Fig. 1.3).

Sample analysis for the presence of CoTS DNA was conducted using standard PCR methods targeting the CoTS mitochondrial cytochrome oxidase subunit 1 gene (mtCOI), specifically a short 126 bp fragment and a long 919 bp fragment, as described above for the first pilot study. PCR was conducted in 20 µl volumes with AmpliTaq Gold master mix (Life Technologies), primer concentrations of 0.4 µM and 1 µl template.

To determine whether fish holding water contained any CoTS DNA, two 2 L seawater samples were collected at each fish collection site at the 1 µm (nominal mesh size) bag filtration outlet for subsequent CoTS DNA analyses. A total of 16 such water samples were taken throughout this pilot study and tested for the presence of CoTS DNA using the large 919bp fragment primers. Despite this pilot study being conducted outside the CoTS spawning season to prevent contamination with CoTS larvae, CoTS DNA was detected in samples from five of the eight collection locations. This clearly compromised the faecal samples collected from fish kept overnight in this filtered seawater during this pilot study, at least at these five collection locations.

To confirm whether the spines observed in faeces from Spangled emperor and Starry pufferfish were from the Pacific Crown-of-Thorns Starfish (*Acanthaster* cf. *solaris*), a few spines were isolated from each faecal sample and evaluated for CoTS using both the small 126bp and large 919bp fragment primer sets. Bands in the 919bp large fragment assay demonstrated these spines were from CoTS, although contamination from filtered seawater containing CoTS DNA cannot be ruled out.

Across the 162 faecal and gut content samples, CoTS DNA was detected using the large 919bp fragment primer sets in a total of six fish species. These six samples positive for CoTS DNA (919bp) were collected at locations that were also positive for CoTS DNA (919bp). We cannot rule out that the positive detection of CoTS DNA in these six faecal samples is not due to contamination by filtered seawater containing CoTS DNA. However, if filtered seawater used to hold the fish overnight contained CoTS DNA and this water cross contaminated fish faecal samples, the expectation would be that all faecal samples collected at these five collection locations would return a positive result for CoTS DNA. This was not the case. Regardless, as contamination cannot be ruled out, these six samples that tested positive for CoTS DNA were not further considered in the findings of our overall study.

Across the 162 faecal and gut content samples, CoTS DNA was detected using the small 126bp fragment primer sets in a large number of faecal samples. This result raised concern of contamination as it was unexpected that so many samples could be positive for CoTS DNA. Further testing with the small 126bp fragment primers revealed that contamination was indeed present, however the specific source was indeterminable, rather it appeared to be originating from the pipettes and bench space. It was determined through significant experimental troubleshooting that the contamination was the 126bp PCR product rather than CoTS DNA. A clean work flow was re-established and the samples tested again using the small 126bp fragment primers. The large number of samples containing the 126bp amplicon was confirmed and it is likely that a significant proportion of the samples were inadvertently contaminated with the 126bp fragment. How this contamination occurred is still unclear, however, it made interpretation of results using the small 126bp fragment primers impossible for this pilot study.

In conclusion, this second pilot study demonstrated the need for further refinement of collection, preservation and analyses methods, to prevent potential contamination of fish faecal and gut content samples ^10^ including from CoTS DNA present in the environment ^1,2^. These methods were developed and implemented as described below, for collection and preservation (Supplementary Text S2) and preventing contamination (Supplementary Text S3) of faecal and gut content samples. The laboratory analyses methods applied to faecal and gut content samples are described in detail in the Materials and Methods section of the main text.


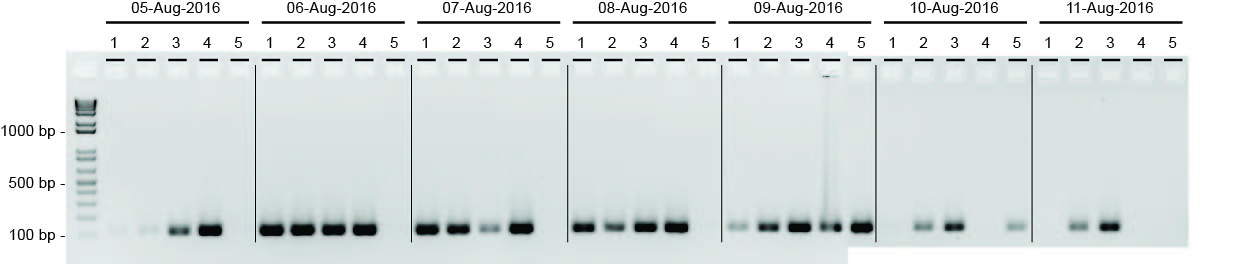


## Fig. 1.1 *Acanthaster* cf. *solaris* DNA in faecal samples from Blackspotted Puffer. Detection of Pacific Crown-of-Thorns Starfish (CoTS, *Acanthaster* cf. *solaris*) mtCOI gene fragment in faeces collected from five Blackspotted Puffers (*Arothron nigropunctatus*) up to seven days post-feeding of freshly-killed CoTS. Numbers 1 through to 5 indicate the experimental fish and the dates are consecutive days post-feeding of CoTS.


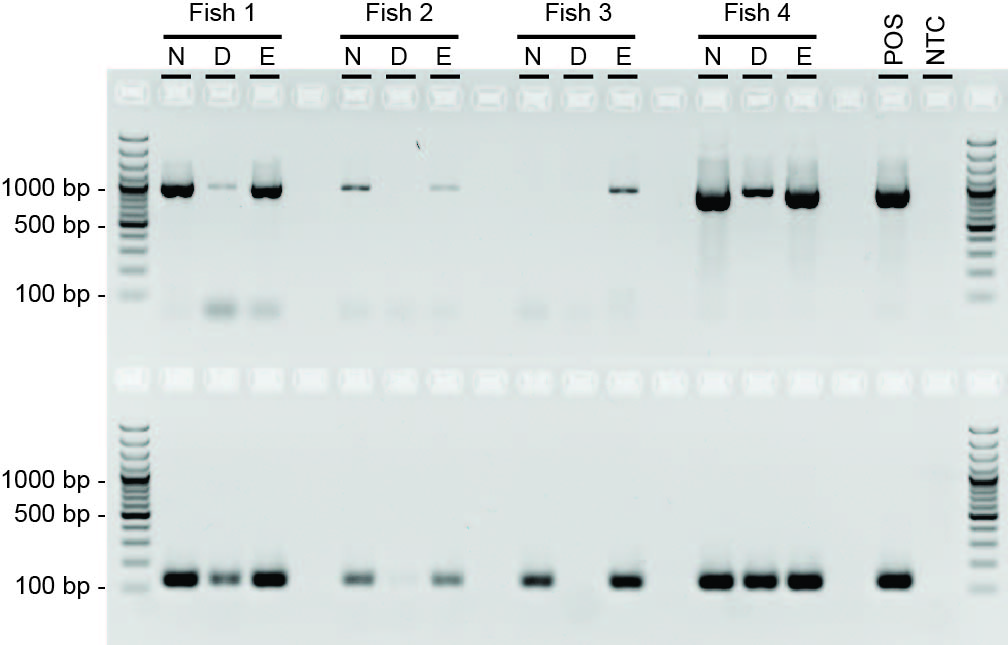


## Fig. 1.2 Preservation methods for fish faecal samples. Effect of sample preservation method on amplification of Pacific Crown-of-Thorns Starfish (CoTS, *Acanthaster* cf. *solaris*) mtCOI gene fragments. Faecal samples from four Blackspotted Puffers (*Arothron nigropunctatus*) were stored in liquid nitrogen (N), dimethylsulfoxide (D) or 100% ethanol (E). The top panel is a PCR of the 919 bp CoTS mtCOI fragment and the lower panel is a PCR of the 126 bp CoTS mtCOI fragment. POS = positive control and NTC = no template control.


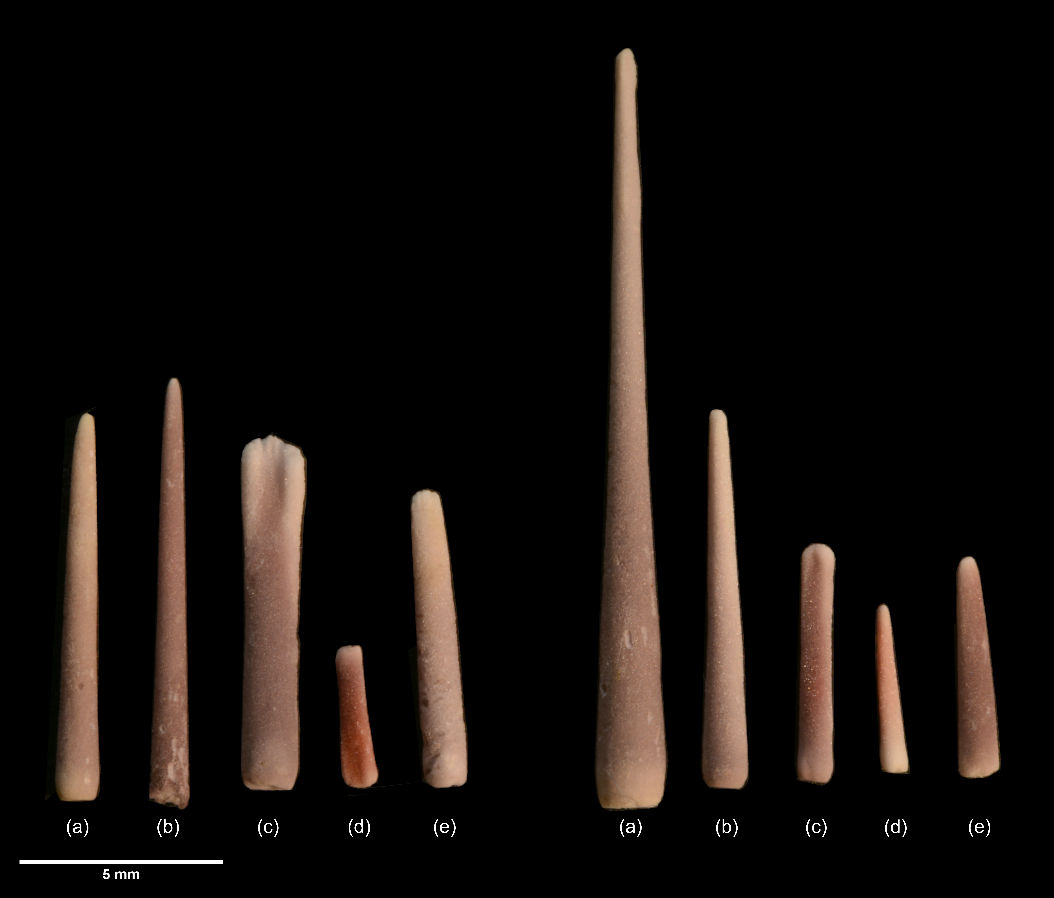

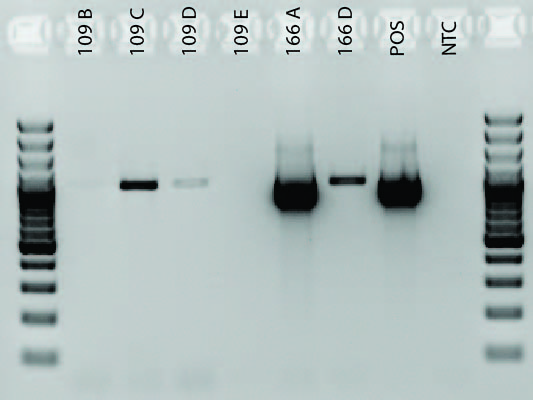

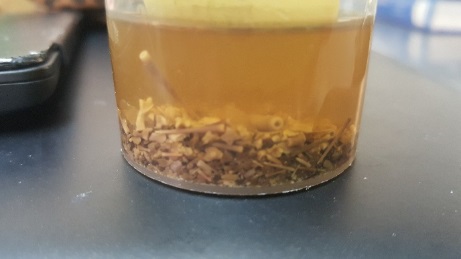

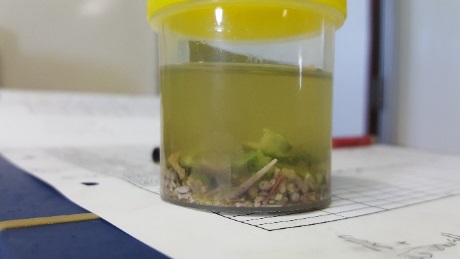


## Fig. 1.3 *Acanthaster* cf. *solaris* spines in faecal samples from wild-caught fish. Spines from the Pacific Crown-of-Thorns Starfish *Acanthaster* cf. *solaris* detected in faeces from wild-caught Spangled Emperor (*Lethrinus nebulosus*) and Starry Puffer (*Arothron stellatus*). Left panel shows the two faecal samples (top: *L. nebulosus*; bottom: *A. stellatus*). Mid panel shows detailed montage of selected spines in these two samples, showing morphologies confirming *Acanthaster* spp. origin namely (a) primary aboral spine, (b) latero-oral spine, (c) oral spine, (d) subambulacral spine, and (e) circumoral spine ^3^ (left: *L. nebulosus*; right: *A. stellatus*). Right panel shows results from PCR analysis for the presence of the large 919bp CoTS mtCOI fragment on selected spines from the two faecal samples (109: *L. nebulosus*; 166: *A. stellatus*). POS = positive PCR control; NTC = negative PCR control.

# Text 2. Collection and preservation of faeces and gut contents from coral reef fish species

All field collections of coral reef fish were conducted with permission from the GBRMPA, the Qld DAF, and the JCU AEC.

## First field trip – January 2018

Potential predation by fish on early CoTS life history stages (gametes, planktonic larvae and newly settled juveniles) was examined by collecting a range of fish species on four midshelf reefs experiencing CoTS population outbreaks (Fig. 1, Table 2) from the RV Cape Ferguson during the CoTS spawning season (02^nd^ to 12^th^ January 2018) as follows. Fish were collected on SCUBA or snorkel at 1 to 9 m depth using either fence nets and/or hand nets and diluted clove oil solution ^8,9^ following JCU AEC Standard Operating Procedures (SoP numbers AQU-008 and AQU-012), or a baited hook and fishing line. Following capture, fish were transported back to the vessel in holding buckets (22 L) or plastic crates (68 L) filled with aerated seawater and covered with lids.

To prevent contamination of faecal samples with CoTS DNA present in seawater during the CoTS spawning season ^2^, subsequent fish processing and holding on the vessel’s back deck was conducted using ultrafiltered (UF) seawater (nominal 0.04 µm) from the National Sea Simulator brought onto the RV Cape Ferguson for this trip. On the vessel, individual fish were captured from holding buckets or plastic crates using a hand net, blotted dry and rinsed successively in three separate 22 L washing buckets filled with UF seawater to remove any potential CoTS DNA on the external surfaces of the fish. Washing buckets and associated hand nets were decontaminated with 10% bleach for at least 30 min and rinsed with UF seawater prior to use; all three washing buckets and associated hand nets were replaced after rinsing 20 fish. Following rinsing, individual fish were transferred into a new plastic bag partly filled with UF seawater, completely filled with 100% O_2_, closed with rubber bands, and kept overnight. Individual bags were placed within a larger plastic bag containing freshwater from the RV Ferguson as a CoTS DNA contamination buffer; the vessel’s freshwater supply is a combination of town water and desalinated (<1 µm) seawater. To control temperature within individual bags, this larger plastic bag was placed in a large circular holding tank (450 L) or plastic crate (68 L) with flow-through ambient saltwater and shielded by a large cover or plastic crate lid. All buckets, plastic crates, holding tanks, and lids were decontaminated with 10% bleach for at least 30 min and rinsed using UF seawater prior to subsequent use on the back deck.

The next morning the contents of individual bags were filtered over a 40 µm mesh sieve, carefully rolling up the top of the bags to avoid freshwater dripping into the bag or onto the mesh sieve. Plastic bags were only used once for faeces collection. The presence or absence of faecal material, including potential CoTS material, was noted for each fish. Individual fish were measured (Standard Length, SL, in mm) and placed into a bucket filled with aerated seawater for return to the site of collection (see Supplementary Material, video). Material in the mesh sieve was concentrated by either direct or indirect transfer into a sample preservation vial. For direct transfer, UF seawater was used to concentrate the sample to an ‘edge’ of the mesh sieve and transferred into a 5 ml sample vial using 100% EtOH. The vial was then topped up with 100% EtOH. For indirect transfer, UF seawater was used to transfer the sample onto a 47 mm Ø filter disc cut from a 40 µm nylon mesh placed on a filter holder within a filter funnel clamped to the set-up. The sample was subsequently passed through this filter disc under vacuum to simultaneously capture the faeces and remove seawater. The filter disc was removed from the filter holder and folded with the sample side on the inside using forceps, and transferred into a 5 ml preservation vial topped up with 100% EtOH. Where required, metal spatulas or forceps were used to concentrate and/or transfer material from mesh sieves. Prior to use, all mesh sieves, filter holders, filter funnels, clamps, spatulas, and forceps were decontaminated with 10% bleach for at least 30 min and rinsed with reverse osmosis (RO) water.

## Second field trip – July 2018

Potential predation by fish on settled CoTS life history stages (juveniles, sub-adults and adults) was examined by collecting a range of fish species on five midshelf reefs experiencing various levels of CoTS population outbreaks (Fig. 1, Table 2) from the RV Cape Ferguson outside the CoTS spawning season (02^nd^ to 12^th^ July 2018) as follows. Fish were collected on SCUBA or snorkel at 1 to 9 m depth using either fence nets and/or hand nets, or a baited hook and fishing line, or from the vessel and tenders using a baited hook and fishing line. Fish that showed signs of barotrauma (i.e. inflated swim bladder) were vented with a hollow hypodermic needle following JCU AEC Standard Operating Procedures (SoP number AQU-020). Following capture, fish were transported back to the vessel in buckets or plastic crates filled with aerated seawater. Fish returned to or captured on the vessel were kept in large circular holding tanks with flow-through ambient seawater to maintain dissolved oxygen (DO) levels above 90% and minimize build-up of ammonia until processing.

To prevent contamination of faecal samples with CoTS DNA present in seawater outside the CoTS spawning season ^1^, subsequent fish processing and holding on the vessel’s back deck was conducted using UF seawater obtained using a custom-designed multi-stage filtration system. The system comprised of three subsequent stages of treatment for unfiltered seawater to remove CoTS DNA, namely (i) mechanical filtration through pressurised bag filter (Waterco C50) with 1 µm nominal mesh size, (ii) mechanical filtration through an ultra-filtration unit (Pentair) with a nominal membrane pore size of 0.02 µm, and (iii) UV-C exposure through a ultra-violet disinfection unit (Tropical Marine HF Pro 55W 240V) at an estimated minimum dose of 40 mW cm^-2^ sec^-1^ (Fig. 2.1). Prior to deployment in the field, various combinations of mechanical filtration and UV-C exposure were tested (Table 2.3), with the combination three subsequent stages of treatment yielding the best results for CoTS DNA removal.

To process and hold fish for faecal sample collection, individual fish were captured from the holding tank using a hand net, blotted dry, measured (SL, mm), and rinsed successively in three separate 22 L washing buckets (smaller fish), or three separate 68 L plastic crates (larger fish), filled with UF seawater to remove any potential CoTS DNA clinging to the fish. Washing buckets and plastic crates, and associated hand nets were decontaminated with 10% bleach for at least 30 min and rinsed with UF seawater prior to use. All three washing buckets and associated hand nets were replaced after rinsing ten fish; all three plastic crates and associated hand nets were replaced after rinsing five fish. Following rinsing, individual fish were transferred into a 22 L bucket, 58 L plastic crate, or 450 L circular holding tank filled with UF seawater and continuously aerated with individual air stones, covered with lids, and kept overnight. All buckets, plastic crates, holding tanks, lids, air stones, and air hoses were decontaminated with 10% bleach for at least 30 min and rinsed with UF seawater prior to subsequent use.

The next morning fish were removed from their bucket, plastic crate or holding tank using a decontaminated hand net for each fish, and either returned to the reef directly (for fish collected from the main vessel) or placed into one of the holding tanks with flow-through ambient seawater and covered with a lid for subsequent return to the site of collection. The contents of individual buckets, plastic crates or holding tanks were filtered over a 40 µm mesh sieve, and the presence or absence of faecal material, including potential CoTS material, was noted for each fish. Material in the mesh sieve was concentrated by either direct or indirect transfer into 5 ml or 50 ml preservation vial depending on the amount of material, as described above for the field trip in January 2018. Prior to use, all hand nets, mesh sieves, filter holders, filter funnels, clamps, spatulas, and forceps were decontaminated with 10% bleach for at least 30 min and rinsed with reverse osmosis (RO) water.

During both January and July 2018 field trips, measurements of seawater temperature (T, in ⁰C) and DO (in mg l^-1^) were taken in the morning from randomly selected bags, buckets, plastic crates or holding tanks using a HACH HQ40D portable meter (Luminescent Dissolved Oxygen (LDO) probe LDO101). This enabled confirmation of maintaining quality of seawater in which individual fish were held overnight. To avoid cross-contamination from the LDO probe, the seawater in which the fish was held overnight was filtered through a 40 µm mesh sieve (for faeces collection) into a small bucket and temperature and DO were subsequently measured. Care was taken not to stir up the seawater so as to minimise inadvertent increases in DO.

## Third field trip – July 2019

Key fish species identified as potential predators of settled CoTS life history stages (juveniles, sub-adults and adults) could not be collected in sufficient numbers, or at all, using non-lethal methods during the July 2018 field trip. Hence, an additional field trip was conducted during with a range of fish species were collected using spearfishing on four midshelf reefs experiencing various levels of CoTS population outbreaks (Fig. 1, Table 2) from the RV Cape Ferguson outside the CoTS spawning season (12^nd^ to 18^th^ July 2019) as follows. Fish were collected while snorkeling at a maximum of 10 m depth using spearguns without powerhead and immediately euthanized using pithing. Following capture, fish were kept in individual plastic bags on a seawater ice slurry until transport back to the vessel.

To prevent contamination of faecal samples with CoTS DNA present in seawater outside the CoTS spawning season ^1^, subsequent fish processing was conducted as follows. Individual fish were removed from their plastic bag and rinsed with freshwater from the RV Cape Ferguson to remove any potential CoTS DNA clinging to the fish. The gastro-intestinal tract was dissected out and associated contents squeezed into a 50 ml, 120 ml or 500 ml preservation vial depending on the amount of material, and preserved in 100% EtOH. The presence or absence of faecal material, including potential CoTS material, was noted for each fish. Individual fish were subsequently measured (SL, mm) and frozen in individual plastic bags for potential future scientific use. For each dissection, new gloves were donned and all dissecting gear was decontaminated with 10% bleach for at least 30 min and rinsed with RO water prior to use.

## Table S2.1 Collection of faeces and gut contents from coral reef fish species. Coral reef fish species, and number of individuals, collected to examine faeces or gut contents for DNA from the Pacific Crown-of-Thorns Starfish (CoTS, *Acanthaster* cf. *solaris*) during three field trips on the RV Cape Ferguson on the Great Barrier Reef, Australia, in (a) January 2018, (b) July 2018, and (c) July 2019. ^C^ = denotes fish species that served as negative controls. Scientific names, common names and CAAB number from <https://www.cmar.csiro.au/caab/>); CAAB = Codes for Australian Aquatic Biota; SL = Standard Length; S.E. = Standard Error.

Please see Excel file ‘Table S2.1 (Collections)’

## Table 2.2 Diet information for coral reef fish species. Information on diet and feeding preferences for coral reef fish species obtained from the primary scientific and grey literature. Fish species were collected to examine faeces or gut contents for DNA from the Pacific Crown-of-Thorns Starfish (CoTS, *Acanthaster* cf. *solaris*) during three field trips on the RV Cape Ferguson on the Great Barrier Reef, Australia, in January 2018 and July 2018 and 2019. ^C^ = fish species that served as a negative control during these three field trips; ^x^ = fish species reported as feeding on live CoTS in the literature; ^#^ = fish species reported as feeding on injured, moribund or dead CoTS in the literature) (Table 1). Scientific names, common names and CAAB number from <https://www.cmar.csiro.au/caab/>); CAAB = Codes for Australian Aquatic Biota.

| **Species** | **Common name** | **CAAB number** | **Information on diet and food items** | **References** |
| --- | --- | --- | --- | --- |
| **Acanthuridae** | |  |  |  |
| *Acanthurus nigrofuscus^C^* | Dusky Surgeonfish | 37 437014 | Filamentous benthic algae | ^11-13^ |
| *Ctenochaetus striatus^C^* | Lined Bristletooth | 37 437022 | Filamentous algae, sediment and detritus | ^11-13^ |
| **Apogonidae** | |  |  |  |
| *Cheilodipterus quinquelineatus* | Fiveline Cardinalfish | 37 327090 | Planktonic and benthic crustaceans; small fishes | ^11,14^ |
| *Nectamia fusca^1^* | Ghost Cardinalfish | 37 327059 | Planktonic and benthic crustaceans and other invertebrates; small fishes | ^11,14^ |
| **Balistidae** | |  |  |  |
| *Abalistes stellatus* | Starry Triggerfish | 37 465011 | Benthic crustaceans, molluscs and other invertebrates including echinoderms; fishes | ^15^ |
| *Balistapus undulatus^X,#^* | Orangestripe Triggerfish | 37 465047 | Wide range of benthic invertebrates including echinoderms (e.g. sea urchin *Echinometra mathaei, Diadema* spp.*)* | ^13,16-18^ |
| *Sufflamen chrysopterum^2^* | Eye-stripe Triggerfish | 37 465078 | Benthic and planktonic invertebrates, including sea urchins (*Diadema* spp.) | ^18^ |
| **Caesionidae** | |  |  |  |
| *Caesio cuning* | Yellowtail Fusilier | 37 346018 | Primarily zooplankton | ^19^ |
| **Chaetodontidae** | |  |  |  |
| *Chaetodon aureofasciatus^#^* | Goldstripe Butterflyfish | 37 365013 | Hard-corals and non-coral macro-invertebrates | ^19,20^ |
| *Chaetodon baronessa^C, #^* | Triangular Butterflyfish | 37 365034 | Almost exclusively hard-corals | ^11,19,20^ |
| *Chaetodon citrinellus^#^* | Citron Butterflyfish | 37 365036 | Hard-corals, sea anemones and other benthic invertebrates | ^11,19,20^ |
| *Chaetodon kleinii^#^* | Klein's Butterflyfish | 37 365040 | Hard-corals, sea anemones and other benthic invertebrates | ^11,20^ |
| *Chaetodon lineolatus^#^* | Lined Butterflyfish | 37 365041 | Sea anemones, other benthic invertebrates; non-coral | ^11,19,20^ |
| *Chaetodon melannotus^C^* | Blackback Butterflyfish | 37 365043 | Mainly hard- and soft-corals | ^11,19,20^ |
| *Chaetodon rainfordi^C,#^* | Rainford's Butterflyfish | 37 365053 | Almost exclusively hard-corals | ^19,20^ |
| *Chaetodon trifascialis^C^* | Chevron Butterflyfish | 37 365058 | Almost exclusively hard-corals | ^11,19-21^ |
| *Chaetodon trifasciatus^C^* | [a butterflyfish] | 37 365094 | Almost exclusively hard-corals | ^11,19^ |
| *Chaetodon vagabondus^#^* | Vagabond Butterflyfish | 37 365062 | Sea anemones, other benthic invertebrates | ^11,19,20^ |
| *Chelmon rostratus* | Beaked Coralfish | 37 365017 | Benthic crustaceans, worms and other invertebrates; non-coral | ^19,20^ |
| **Haemulidae** | |  |  |  |
| *Diagramma pictum labiosum* | Painted Sweetlips | 37 350003 | Benthic crustaceans, molluscs and echinoderms; fishes | ^15,19,22,23^ |
| *Plectorhinchus chaetodonoides* | Spotted Sweetlips | 37 350014 | Benthic crustaceans, molluscs and fishes | ^24^ |
| *Plectorhinchus picus* | Dotted Sweetlips | 37 350023 | Benthic crustaceans and molluscs | ^15^ |
| **Labridae** | |  |  |  |
| *Cheilinus chlorourus* | Floral Maori Wrasse | 37 384064 | Benthic, hard-shelled echinoderms, crustaceans and molluscs | ^11,13^ |
| *Cheilinus fasciatus^#^* | Redbreast Maori Wrasse | 37 384066 | Benthic, hard-shelled crustaceans, molluscs and echinoderms; sea urchin predator (e.g. *Diadema* spp.) | ^13,18^ |
| *Cheilinus trilobatus* | Tripletail Maori Wrasse | 37 384044 | Benthic, hard-shelled echinoderms, crustaceans and molluscs; sea urchin predator (e.g. *Echinometra mathaei*) | ^11,13,16,25^ |
| *Cheilinus undulatus^x^* | Humphead Maori Wrasse | 37 384038 | Benthic, hard-shelled molluscs, echinoderms and crustaceans; fishes | ^13,26^ |
| *Choerodon fasciatus* | Harlequin Tuskfish | 37 384073 | Benthic, hard-shelled crustaceans, molluscs and echinoderms | ^13^ |
| *Choerodon schoenleinii* | Blackspot Tuskfish | 37 384010 | Benthic, hard-shelled crustaceans, molluscs and echinoderms; sea urchin predator (*Echinometra mathaei*) | ^13,17^ |
| *Cirrhilabrus punctatus* | Finespot Wrasse | 37 384083 | Planktivorous | ^27^ |
| *Coris aygula* | Redblotched Wrasse | 37 384090 | Benthic, hard-shelled molluscs, crustaceans and echinoderms; sea urchin predator (e.g. *Echinometra mathaei*) | ^11,13,16,25^ |
| *Coris batuensis^3^* | Variegated Wrasse | 37 384098 | Mostly benthic crustaceans and molluscs | ^11^ |
| *Coris gaimard* | Clown Wrasse | 37 384094 | Benthic, hard-shelled crustaceans, molluscs and echinoderms*;* sea urchin predator (e.g. *Echinometra mathaei*) | ^13,16,25,28^ |
| *Epibulus insidiator* | Slingjaw Wrasse | 37 384104 | Benthic crustaceans and small fishes | ^11,13^ |
| *Gomphosus varius* | Birdnose Wrasse | 37 384106 | Benthic crustaceans, molluscs and other small invertebrates | ^11,13^ |
| *Halichoeres marginatus* | Dusky Wrasse | 37 384114 | Mostly wide range of benthic invertebrates | ^11^ |
| *Halichoeres melanurus^#^* | Hoeven's Wrasse | 37 384032 | Mostly wide range of benthic invertebrates; also algae | ^11^ |
| *Halichoeres trimaculatus* | Threespot Wrasse | 37 384122 | Mainly benthic crustaceans and molluscs | ^11^ |
| *Hemigymnus fasciatus* | Fiveband Wrasse | 37 384124 | Mainly benthic crustaceans, also molluscs and echinoderms | ^11,13^ |
| *Hemygymnus melapterus* | Thicklip Wrasse | 37 384125 | Mainly benthic crustaceans and molluscs; also polychaetes and echinoderms | ^11,13^ |
| *Hologymnosus doliatus* | Pastel Slender Wrasse | 37 384127 | Benthic crustaceans, echinoderms and polychaetes; small fishes | ^13^ |
| *Novaculichthys taeniourus* | Carpet Wrasse | 37 384140 | Benthic, hard-shelled molluscs, echinoderms and crustaceans; also polychaetes | ^13^ |
| *Oxycheilinus diagrammus^4,#^* | Violetline Maori Wrasse | 37 384065 | Mainly crustaceans and fishes | ^13,29^ |
| *Pseudocheilinus hexataenia* | Sixline Wrasse | 37 384143 | Mainly benthic crustaceans, also small molluscs | ^11^ |
| *Thalassoma amblycephalum* | Bluehead Wrasse | 37 384164 | Planktonic crustaceans | ^11,13^ |
| *Thalassoma jansenii^#^* | Jansen's Wrasse | 37 384166 | Mostly wide range of benthic invertebrates | ^11^ |
| *Thalassoma lunare^#^* | Moon Wrasse | 37 384167 | Benthic crustaceans and wide range of other invertebrates; fish and gastropod eggs | ^11,13,29^ |
| **Lethrinidae** | |  |  |  |
| *Gymnocranius audleyi* | Collar Seabream | 37 351018 | Benthic crustaceans, molluscs, annelids and other invertebrates | ^19,30^ |
| *Gymnocranius euanus* | Paddletail Seabream | 37 351022 | Mainly benthic molluscs and echinoderms | ^15,19,30^ |
| *Gymnocranius grandoculis* | Robinson's Seabream | 37 351005 | Mainly benthic molluscs; also echinoderms, worms and small fishes | ^15,19,30^ |
| *Lethrinus atkinsoni^x,#^* | Yellowtail Emperor | 37 351013 | Mainly benthic crustaceans, molluscs and echinoderms; fishes; sea urchin predator (*Echinometra mathaei*) | ^15,17,19,30^ |
| *Lethrinus lentjan* | Redspot Emperor | 37 351007 | Mainly benthic crustaceans, molluscs and echinoderms; also polychaetes and fishes | ^15,19,22,30,31^ |
| *Lethrinus miniatus^5,x,#^* | Redthroat Emperor | 37 351009 | Benthic crustaceans, echinoderms, and molluscs; fishes | ^15,19,23,30,32,33^ |
| *Lethrinus nebulosus ^x,#^* | Spangled Emperor | 37 351008 | Benthic crustaceans, echinoderms, and molluscs; fishes | ^15,19,22,30,33^ |
| *Lethrinus obsoletus* | Orangestriped Emperor | 37 351019 | Benthic crustaceans, molluscs, echinoderms and polychaetes | ^15,19,30^ |
| *Lethrinus ornatus* | Ornate Emperor | 37 351015 | Benthic crustaceans, molluscs, echinoderms and polychaetes; small fishes | ^19,30^ |
| *Lethrinus rubrioperculatus* | Spotcheek Emperor | 37 351012 | Mostly benthic crustaceans, echinoderms and molluscs; fishes | ^15,19,30^ |
| *Monotaxis grandoculis^x^* | Bigeye Seabream | 37 351026 | Benthic crustaceans, molluscs, echinoderms and polychaetes | ^15,19,30^ |
| **Lutjanidae** | |  |  |  |
| *Lutjanus adetti* | Hussar | 37 346033 | Benthic crustaceans and other invertebrates including echinoderms; fishes | ^15^ |
| *Lutjanus bohar^#^* | Red Bass | 37 346029 | Mainly fishes; also benthic crustaceans, molluscs and other invertebrates | ^15,19^ |
| *Lutjanus fulviflamma* | Blackspot Snapper | 37 346034 | Mainly small fishes and benthic crustaceans | ^11,15,19,29^ |
| *Lutjanus gibbus^#^* | Paddletail | 37 346028 | Benthic crustaceans and other invertebrates including echinoderms; fishes | ^15,19^ |
| *Lutjanus quinquelineatus* | Fiveline Snapper | 37 346006 | Benthic crustaceans and other invertebrates including echinoderms; fishes | ^15^ |
| *Lutjanus russelli* | Moses' Snapper | 37 346065 | Mainly fishes and benthic crustaceans | ^15,22^ |
| *Lutjanus sebae* | Red Emperor | 37 346004 | Mainly fishes and benthic crustaceans; also echinoderms | ^15,19,22,33^ |
| *Symphorus nematophorus* | Chinamanfish | 37 346017 | Mainly fishes and benthic crustaceans | ^15^ |
| **Microdesmidae** | |  |  |  |
| *Ptereleostris evides* | Arrow Dartgoby | 37 435015 | Small planktonic crustaceans | ^11^ |
| **Monacanthidae** | |  |  |  |
| *Oxymonacanthus longirostris^C^* | Harlequin Filefish | 37 465062 | Almost exclusively hard-corals (*Acropora* spp) | ^11,34^ |
| *Paraluteres prionurus* | Blacksaddle Filefish | 37 465063 | Small benthic invertebrates, eggs; benthic algae | ^24^ |
| **Mullidae** | |  |  |  |
| *Parupeneus multifasciatus^#^* | Banded Goatfish | 37 355026 | Mainly benthic crustaceans; also molluscs and small fishes | ^19^ |
| **Nemipteridae** | |  |  |  |
| *Scolopsis bilineatus^#^* | Two-line Monocle Bream | 37 347031 | Mainly benthic polychaetes and other marine worms; also crustaceans, molluscs and small fishes | ^19,35^ |
| *Scolopis margaritifera* | Pearly Monocle Bream | 37 347033 | Mainly benthic crustaceans, polychaetes, molluscs and small fishes | ^19^ |
| *Scolopsis monogramma* | Rainbow Monocle Bream | 37 347006 | Mainly benthic crustaceans, annelids, molluscs, polychaetes; small fishes | ^19,22^ |
| **Plesiopidae** | |  |  |  |
| *Assessor macneilli* | Blue Scissortail | 37 316004 | Small fishes, crabs, shrimps, copepods, amphipods, gastropods and polychaetes | ^23^ |
| **Pomacanthidae** | |  |  |  |
| *Pomacanthus sexstriatus^#^* | Sixband Angelfish | 37 365010 | Sponges, tunicates, and other invertebrates | ^13^ |
| **Pomacentridae** | |  |  |  |
| *Acanthochromis polyacanthus^x,#^* | Spiny Puller | 37 372015 | Mainly planktivorous (zooplankton) | ^36^ |
| *Amblyglyphidodon curacao^x,#^* | Staghorn Damsel | 37 372017 | Filamentous algae, planktonic crustaceans, wide variety of other small invertebrates; fish and invertebrate eggs | ^11^ |
| *Chromis viridis^6,x,#^* | Blue-green Puller | 37 372053 | Filamentous algae, planktonic crustaceans, wide variety of other small invertebrates; fish and invertebrate eggs | ^11,19^ |
| *Dascyllus aruanus ^x^* | Banded Humbug | 37 372073 | Filamentous algae, planktonic crustaceans, benthic invertebrates; fish and invertebrate eggs | ^11,19^ |
| *Dascyllus reticulatus^#^* | Headband Humbug | 37 372074 | Planktivorous (zooplankton, including fish eggs), also benthic algae and crustaceans | ^24^ |
| *Dischistodus melanotus^C,#^* | Blackvent Damsel | 37 372077 | Benthic algae | ^24^ |
| *Neoglyphidodon melas^7,#^* | Black damsel | 37 372084 | Zooplankton, benthic invertebrates and algae; fish and invertebrate eggs | ^11^ |
| *Neopomacentrus azysron ^x^* | Yellowtail Demoiselle | 37 372087 | Planktivorous (zooplankton) | ^24^ |
| *Pomacentrus amboinensis ^x^* | Ambon Damsel | 37 372106 | Filamentous algae, planktonic crustaceans, other small invertebrates; fish and invertebrate eggs | ^11^ |
| *Pomacentrus bankanensis* | Speckled Damsel | 37 372108 | Primarily filamentous algae, also copepods and other small invertebrates; fish and invertebrate eggs | ^11^ |
| *Pomacentrus chrysurus^8,#^* | Whitetail Damsel | 37 372110 | Primarily filamentous algae, also various small invertebrates; fish and invertebrate eggs | ^11^ |
| *Pomacentrus coelestis* | Neon Damsel | 37 372111 | Mainly planktivorous (zooplankton) | ^19,37^ |
| *Pomacentrus moluccensis ^x,#^* | Lemon Damsel | 37 372118 | Filamentous algae and wide variety of small invertebrates; fish and invertebrate eggs | ^19^ |
| *Pomacentrus magniseptus* | a damselfish | 37 372156 | Planktivorous (zooplankton), benthic algae | ^24^ |
| *Pomacentrus wardi^C,#^* | Ward's Damsel | 37 372127 | Mainly benthic algae; also soft coral | ^38^ |
| **Scaridae** | |  |  |  |
| *Scarus flavipectoralis^C^* | Yellowfin Parrotfish | 37 386014 | Benthic algae | ^24^ |
| **Serranidae** | |  |  |  |
| *Chromileptes altivelis* | Barramundi Cod | 37 311044 | Fishes and crustaceans | ^11,39^ |
| *Epinephelus cyanopodus* | Purple Rockcod | 37 311145 | Mainly benthic fishes and crustaceans | ^15,40^ |
| *Epinephelus fasciatus* | Blacktip Rockcod | 37 311014 | Benthic crustaceans, fishes and echinoderms | ^15,40^ |
| *Epinephelus polyphekadion* | Camouflage Grouper | 37 311047 | Mainly crustaceans and fishes; also molluscs and echinoderms | ^15,40^ |
| *Plectropomus leopardus^C^* | Common Coral Trout | 37 311078 | Benthic crustaceans (juveniles); fishes (adults) | ^15,40,41^ |
| **Siganidae** | |  |  |  |
| *Siganus corallinus^C^* | Coral Rabbitfish | 37 438008 | Benthic algae (mostly red and green macroalgae) | ^11,13,42^ |
| **Tetraodontidae** | |  |  |  |
| *Arothron nigropunctatus^9,#^* | Blackspotted Puffer | 37 467027 | Corals (usually *Acropora* tips) and sea anemones; also algae and crustaceans | ^11^ |
| *Arothron stellatus^x^* | Starry Puffer | 37 467014 | Benthic crustaceans, molluscs and echinoderms | ^15^ |
| *Canthigaster papua* | Netted Toby | 37 467042 | Primarily filamentous green and red algae, and coralline red algae; also corals and benthic invertebrates including echinoderms | ^39^ |
| *Canthigaster valentini* | Blacksaddle Toby | 37 467043 | Mainly filamentous green and red algae, and tunicates; also corals and benthic invertebrates including echinoderms and brown and coralline red algae | ^39^ |
| **Tripterygiidae** | |  |  |  |
| *Helcogramma vulcanum* | Volcano Triplefin | n/a | Small benthic invertebrates | ^39^ ^24^ |

Numbers in superscript denote synonyms used in literature reviewed: ^1^ = *Apogon guamensis*, *Apogon nubilus*; ^2^ = *Hemibalistes chrysopterus*; ^3^ = *Coris variegata* (misapplied) ^4^ = *Cheilinus diagrammus*; ^5^ = *Lethrinus chrysostomus*; ^6^ = *Chromis caerulea;* 7 = *Paraglyphidodon melas*; ^8^ = *Pomacentrus flavicauda*; ^9^ = *Tetraodon nigropunctatus.*

## Table 2.3 Removal of CoTS DNA from seawater. Prior to the second field trip in July 2018, mechanical filtration and UV-C exposure were tested alone or in combination for removal of CoTS DNA in seawater. This trial utilised an extremely highly CoTS density, with 27 adult CoTS kept in a 10,000 L tank at the National Sea Simulator in Townsville, Australia. Treatment of water from this tank included a combination of mechanical filtration through two filtration units with a nominal mesh size of 0.1 µm (Sawyer Mini) and a nominal membrane pore size of 0.02 µm (Pentair), and UV-C exposure through a ultra-violet disinfection unit (Tropical Marine HF Pro 55W 240V) at an estimated minimum dose of 40 mW cm^-2^ sec^-1^. The amount of DNA (measured by Qubit dsDNA assay, Thermo) and CoTS specific DNA (as measured by ddPCR) was reduced significantly with mechanical filtration only, and completely removed with the addition of UV treatment (CoTS DNA only). Results presented as the mean and Standard Deviation of triplicate measurements; n.d. = not detected.

| **Test Description** | **DNA concentration**  **(ng L^-1^ sewater)** | **ddPCR CoTS mtCOI (copies L^-1^ seawater)** |
| --- | --- | --- |
| Untreated seawater | 4,894 ± 1,064 | 1,631,813 ± 312,037 |
| Mechanical filtration (0.1 µm and 0.02 µm) | 4 ± 0.4 | 75 ± 106 |
| Mechanical filtration (0.1 µm and 0.02 µm) and UV-C exposure | 6 ± 1.2 | n.d. |
| Fresh water | 3 ± 3 | n.d. |


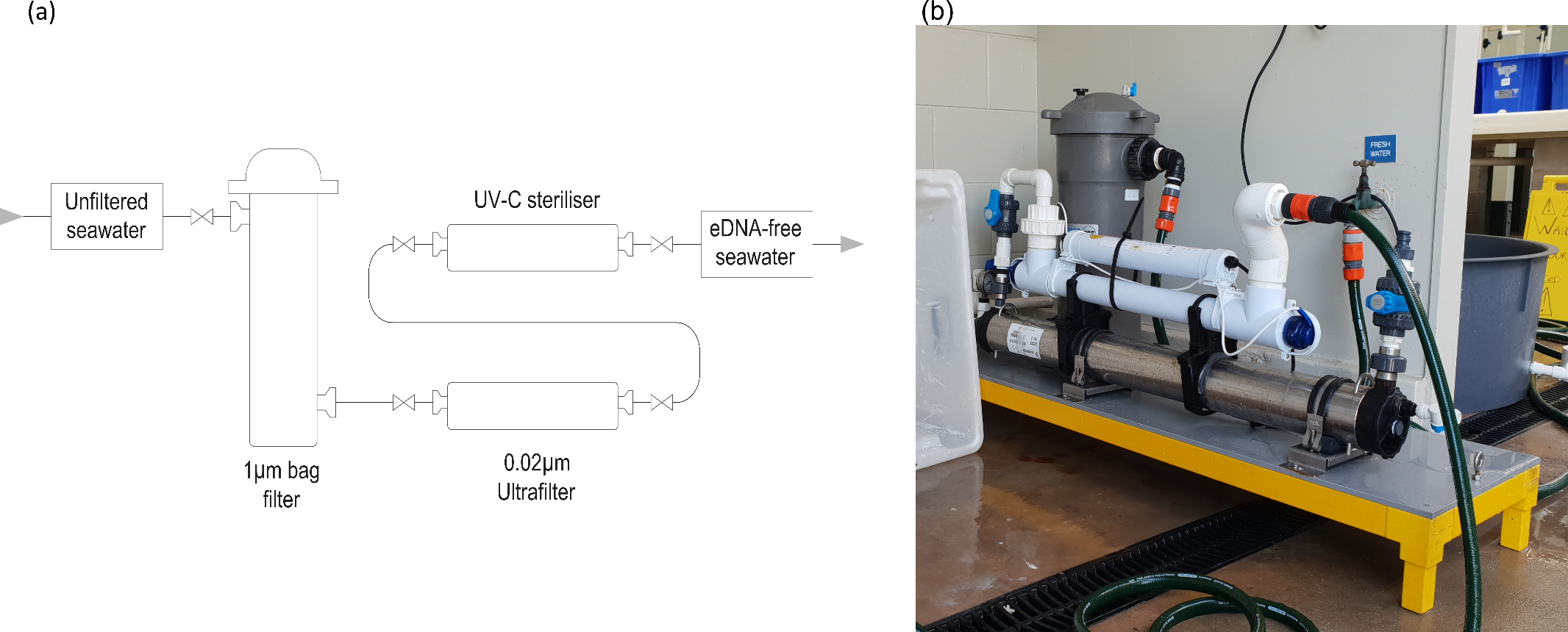


## Fig. 2.1 Removal of CoTS DNA from raw seawater. The system, presented as (a) schematic and (b) in reality, comprised of three subsequent stages of treatment for unfiltered seawater to remove CoTS DNA, namely (i) mechanical filtration through pressurised bag filter (Waterco C50) with 1 µm nominal mesh size, (ii) mechanical filtration through an ultra-filtration unit (Pentair) with a nominal membrane pore size of 0.02 µm, and (iii) UV-C exposure through a ultra-violet disinfection unit (Tropical Marine HF Pro 55W 240V) at an estimated minimum dose of 40 mW cm^-2^ sec^-1^.

# Text S3. Preventing contamination of faecal and gut content samples

To prevent and examine potential contamination of fish faecal and gut content samples from CoTS DNA present in the environment ^1,2^, a comprehensive set of negative and positive control measures were taken prior and during field trips (Table 3.1). First, all field collection trips on the central GBR were conducted on reefs that were experiencing CoTS outbreaks (Table 2), with some of these reefs being visited by the CoTS control program run by GBRMPA ^43^. This control program kills individual CoTS by using a single small volume injection of oxbile ^44^; the dead CoTS are left on the reef to decompose. Four days after injection, little evidence of dead CoTS remain except for small piles of spines and skeletal elements ^44^. Similar disintegration rates have been reported for moribund and dead CoTS with few spines and ossicles remaining after 4 to 8 days ^45,46^. Fish predation on these moribund and dead CoTS could greatly increase the probability of detecting CoTS DNA in fish faeces and gut contents when no predation on life CoTS had occurred (i.e. false positive). Further, results from a preliminary study suggests that CoTS DNA decays exponentially in seawater to being undetectable after 8 days (Doyle, unpublished). To minimise the probability of false positives, we closely consulted with GBRMPA and only visited reefs that had either not experienced CoTS culling at all, or had not experienced CoTS culling for at least four weeks prior to the respective field trip. For the latter reefs, this would have allowed ample time for complete disintegration of culled CoTS (4-8 days) and complete decay of associated CoTS DNA in surrounding waters (8 days). For the January 2018 field trip, CoTS had never been culled (18-025) or had not been culled in the five weeks prior (Bramble, Kelso and Rib reefs). Similarly, for the July 2018 field trip, the final CoTS culling on Bramble, Rib, Kelso, Lodestone and 18-025 reefs took place at least five weeks prior (i.e. before or on 29 May 2018), and ceased until after this trip was finished in consultation with GBRMPA. Finally, for the July 2019 field trip, CoTS had not been culled (Kelso, Little and Big Broadhurst reefs) or had not been culled in the four weeks prior (Keeper).

Second, the following set of negative controls were put in place during field work (Table 3.1). These comprised:

- Collection of faeces from fish species that were highly unlikely to consume CoTS such as obligate herbivores, corallivores and piscivores (Table 2.2). Faecal samples were collected and processed as for other fish species described above (Supplementary Material Text S2) and in the Materials and Methods section of the main manuscript;
- Water samples from the various water supplies used for processing, rinsing and holding fish, and for processing faecal samples, namely freshwater, unfiltered seawater, UF seawater (both from SeaSim and skid-filtered seawater), RO water, and fish rinsing buckets or plastic crates. For the latter, water samples were taken from the 3^rd^ bucket or plastic crate after final fish rinses (Supplementary Material Text S2). For each water type, two litre water samples were filtered over a 47 mm 1 µm mixed cellulose ester filter, and the filter folded with the sample side on the inside using forceps and transferred into a 1.5 ml sample vial topped up with 540 µl Qiagen buffer ATL. These samples were extracted and tested for the presence of CoTS DNA using methods described in the Materials and Methods section of the main text.
- Water clinging to fish skin after the 3^rd^ rinse in filtered seawater, and prior to placement in overnight bag, bucket, plastic crate or circular holding tank (Supplementary Material Text S2), was sampled by gently stroking the side of the fish using a cotton bud. The cotton end of the bud was subsequently cut off using scissors and placed into a 5 ml sample vial topped up with 100% EtOH. These samples were extracted and tested for the presence of CoTS DNA using methods described in the Materials and Methods section of the main text.
- A handling control of processing and holding fish overnight was conducted using an inanimate object (i.e. fishing lure). This lure was placed into the holding bucket or bin with fish to be processed, and taken through the same procedures applied to all fish and associated faecal samples (Supplementary Material Text S2). These samples were extracted and tested for the presence of CoTS DNA using methods described in the Materials and Methods section of the main text.
- Similarly, an equipment control of processing and preserving faecal samples was conducted using a decontaminated 40 µm mesh sieve. This mesh sieve was treated as if it contained a faecal sample and processed accordingly (Supplementary Material Text S2). These samples were extracted and tested for the presence of CoTS DNA using methods described in the Materials and Methods section of the main text.
- Faecal samples were collected from a range of fish species on reefs at Lizard Island (reef numbers 14-116a, c, d) during the CoTS spawning season in January 2019 (Table 3.2). The focus on this field trip was on fish species, and individuals of similar sizes, that had tested positive for CoTS DNA on reefs in the central GBR in January and July 2018. Lizard Island had not experienced a CoTS population outbreak for a few years, and while present, numbers of CoTS had been extremely low (pers. comm. Dr Lye Vale, Lizard Island Research Station, January 2019). Hence, if our sample collection, processing and analyses were robust, we would predict the absence of CoTS DNA in the faeces of any of our fish species that had been tested positive previously on reefs with CoTS outbreaks. During the Lizard Island trip, many of the same negative and positive controls described above were also implemented (Table 3.1).

Finally, the following set of positive controls were put in place during field work (Table 3.1). These comprised:

- A plankton sample was collected using an oblique plankton tow at each collection reef to determine the presence of CoTS gametes or planktonic larvae during the CoTS spawning season (January 2018, 2019; ^2^). Plankton samples were concentrated, preserved and extracted following ^5^ and CoTS specific DNA determined following ^1^. Potential CoTS DNA in seawater from settled CoTS (July 2018; ^1^) was checked in raw seawater (see ‘negative controls’ above). These samples were extracted and tested for the presence of CoTS DNA using methods described in the Materials and Methods section of the main text.
- Water samples (1 L) collected from a SeaSim aquarium housing CoTS and taken on-board the RV Cape Ferguson during both January and July 2018 trips. These samples were vacuum filtered onto a 47 mm Ø filter cut from a 40 µm nylon mesh using the same procedures for fish faecal samples as described above (Text S3). The filter was placed into a 5 ml sample vial topped up with 100% EtOH. These samples were extracted and tested for the presence of CoTS DNA using methods described in the Materials and Methods section of the main text.
- Freshly collected spines from live CoTS kept in an aquarium at Lizard Island Research Station were placed into a 5 ml sample vial topped up with 100% EtOH (January 2019). These samples were extracted and tested for the presence of CoTS DNA using methods described in the Materials and Methods section of the main text.

## Table 3.1. Preventing contamination of faecal and gut content samples. Negative and positive control samples collected and processed during field trips, conducted to collect coral reef fish to examine faeces for DNA from the Pacific Crown-of-Thorns Starfish (CoTS, *Acanthaster* cf. *solaris*), on the RV Cape Ferguson (January 2018; July 2018), and from Lizard Island Research Station (January 2019), on the Great Barrier Reef, Australia. No control samples were collected during the July 2019 field trip (see Supplementary Material Text S3 for details). Y = sample collected; N = sample not collected, n/a = sample not applicable; RV CF = RV Cape Ferguson; number of samples collected and processed in between brackets; samples in which CoTS DNA was detected presented in bold.

| **Control types** | **January 2018** | | | | **July 2018** | | | | | **January 2019** |
| --- | --- | --- | --- | --- | --- | --- | --- | --- | --- | --- |
|  | **Bramble** | **Kelso** | **Rib** | **Unnamed** | **Bramble** | **Kelso** | **Lodestone** | **Rib** | **Unnamed** | **Lizard Island** |
|  | **18-029** | **18-030** | **18-032** | **18-025** | **18-029** | **18-030** | **18-078** | **18-032** | **18-025** | **14-116a, c, d** |
| **Negative** |  |  |  |  |  |  |  |  |  |  |
| Herbivorous fish species* | Y (4) | Y (4) | Y (1) | Y (3) | n/a | n/a | n/a | n/a | n/a | n/a |
| Corallivorous fish species* | Y (5) | Y (7) | Y (1) | Y (2) | n/a | n/a | n/a | n/a | n/a | n/a |
| Piscivorous fish species* | n/a | n/a | n/a | n/a | Y (5) | Y (2) | N | **Y (1 of 5)^#^** | Y (5) | Y (10) |
| Freshwater (RV CF) | Y (5) | | | | Y (2) | Y (1) | Y (2) | Y (4) | Y (1) | n/a |
| Filtered freshwater (skid) | n/a | | | | n/a | | | | | Y (8) |
| Raw seawater (RV CF) | n/a | | | | **Y (1 of 2)** | Y (1) | Y (3) | **Y (3 of 4)** | Y (1) | Y (8) |
| Filtered seawater (SeaSim) | Y (7) | | | | n/a | | | | | n/a |
| Filtered seawater (skid) | n/a | | | | Y (2) | Y (1) | Y (2) | Y (4) | Y (1) | Y (8) |
| Filtered seawater (3^rd^ rinse) | N | | | | Y (3) | | | | | Y (14) |
| RO water | Y (7) | | | | N | | | | | N |
| Fish skin (after 3^rd^ rinse) | N | | | | N | Y (2) | Y (4) | Y (6) | Y (2) | Y (7) |
| Handling control (lure) | Y (3) | | | | Y (4) | | | | | Y (8) |
| Equipment control (mesh) | Y (12) | | | | Y (3) | | | | | Y (8) |
| **Positive** |  |  |  |  |  |  |  |  |  |  |
| Plankton tows | **Y (3 of 3)** | Y (3) | **Y (3 of 3)** | Y (3) | n/a | | | | | Y (7) |
| Seawater (SeaSim CoTS aquarium) | N | | | | **Y (5 of 5)** | | | | | N |
| Spines (LIRS CoTS aquarium) | n/a | | | | n/a | n/a | n/a | n/a | n/a | **Y (2 of 2)** |

* Herbivorous fish species: *Acanthurus nigrofuscus*, *Ctenochaetus striatus*, *Dischistodus melanotus*, *Pomacentrus wardi*, *Scarus flavipectoralis*, *Siganus corallinus*; Corallivorous fish species: *Chaetodon baronessa*, *C. melannotus*, *C. rainford*, *C. trifascialis*, *C. trifasciatus*, *Oxymonacanthus longirostris*; Piscivorous fish species: *Plectropomus leopardus* (see Table 2.2 for more detail on dietary information of these species). **^#^**A faecal sample from a single Common Coral Trout (*P. leopardus*; 370 mm standard length), caught at Rib Reef in July 2018, tested positive for CoTS DNA.

## Table 3.2. Preventing contamination of faecal and gut content samples. Fish species and total numbers of individuals collected at Lizard Island Research Station in January 2019. These species, and individuals of similar sizes, had tested positive for CoTS DNA on reefs with CoTS outbreaks in the central GBR in January and July 2018 (see Table 3). Number of faecal samples in which CoTS DNA was detected presented in between brackets. Scientific names, common names and CAAB number from <https://www.cmar.csiro.au/caab/>); CAAB = Codes for Australian Aquatic Biota. SL = Standard Length; S.E. = Standard Error.

| **Fish species** | | | | |  |  |  |
| --- | --- | --- | --- | --- | --- | --- | --- |
| **Family** | **Species** | **Common name** | **CAAB number** | **Total** | **Size (mm, SL)** | | |
|  |  |  |  |  | **Mean ± S.E.** | **Min** | **Max** |
| Balistidae | *Balistapus undulatus* | Orangestripe Triggerfish | 37 465047 | 5 (0) | 29 ± 1 | 25 | 33 |
| Haemulidae | *Diagramma pictum labiosum* | Painted Sweetlips | 37 350003 | 1 (0) | 220 |  |  |
| Labridae | *Cheilinus chlorourus* | Floral Maori Wrasse | 37 384064 | 5 (0) | 162 ± 10 | 130 | 180 |
| Lethrinidae | *Lethrinus nebulosus* | Spangled Emperor | 37 351008 | 5 (0) | 267 ± 23 | 220 | 335 |
| Lutjanidae | *Lutjanus fulviflama* | Blackspot Snapper | 37 346034 | 10 (0) | 171 ± 4 | 152 | 190 |
|  | *Lutjanus russelli* | Moses' Snapper | 37 346065 | 2 (0) | 228 ± 2 | 226 | 230 |
| Pomacentridae | *Acanthochromis polyacanthus* | Spiny Puller | 37 372015 | 10 (0) | 29 ± 1 | 25 | 33 |
|  | *Dascyllus aruanus* | Banded Humbug | 37 372073 | 10 (0) | 33 ± 1 | 27 | 39 |
|  | *Neoglyphidodon melas* | Black damsel | 37 372084 | 10 (0) | 28 ± 2 | 19 | 37 |
|  | *Pomacentrus amboinensis* | Ambon Damsel | 37 372106 | 10 (0) | 25 ± 1 | 20 | 27 |
|  | *Pomacentrus chrysurus* | Whitetail Damsel | 37 372110 | 10 (0) | 37 ± 3 | 26 | 49 |
|  | *Pomacentrus coelestis* | Neon Damsel | 37 372111 | 9 (0) | 37 ± 2 | 27 | 45 |
| Serranidae | *Plectropomus leopardus* | Common Coral Trout | 37 311078 | 10 (0) | 340 ± 13 | 270 | 390 |

# References

1. Uthicke S, Lamare M, Doyle JR. eDNA detection of corallivorous seastar (*Acanthaster cf. solaris*) outbreaks on the Great Barrier Reef using digital droplet PCR. *Coral Reefs* **37**, 1229–1239 (2018).

2. Uthicke S*, et al.* Spawning time of *Acanthaster cf. solaris* on the Great Barrier Reef inferred using qPCR quantification of embryos and larvae: do they know it’s Christmas? *Mar Biol* **166**, 1-10 (2019).

3. Motokawa T. Morphology of spines and spine joint in the crown-of-thorns starfish *Acanthaster planci* (Echinodermata, Asteroida). *Zoomorphology* **106**, 247-253 (1986).

4. Pratchett M, Caballes CF, Rivera-Posada JA, Sweatman HPA. Limits to our understanding and managing outbreaks of Crown-of-Thorn Starfish (*Acanthaster* spp.). *Oceanogr Mar Biol Annu Rev* **52**, 133-200 (2014).

5. Doyle JR, McKinnon AD, Uthicke S. Quantifying larvae of the coralivorous seastar *Acanthaster* cf. *solaris* on the Great Barrier Reef using qPCR. *Mar Biol* **164**, 176 (2017).

6. Uthicke S, Doyle J, Duggan S, Yasuda N, McKinnon AD. Outbreak of coral-eating Crown-of-Thorns creates continuous cloud of larvae over 320 km of the Great Barrier Reef. *Scientific Reports* **5**, 16885 (2015).

7. Deagle BE, Eveson JP, Jarman SN. Quantification of damage in DNA recovery from highly degraded samples - a case study on DNA in faeces. *Frontiers in Zoology* **3**, 11 (2006).

8. Munday PL, Wilson SK. Comparative efficacy of clove oil and other chemicals in anaesthetization of *Pomacentrus amboinensis*, a coral reef fish. *J Fish Biol* **51**, 931-938 (1997).

9. Kroon FJ. The efficacy of clove oil for anaesthesia of eight species of Australian tropical freshwater teleosts. *Limnol Oceanogr Methods* **13**, 463-475 (2015).

10. Thomsen PF, Willerslev E. Environmental DNA – An emerging tool in conservation for monitoring past and present biodiversity. *Biol Conserv* **183**, 4-18 (2015).

11. Sano M, Shimizu M, Nose Y. Food habits of teleostean reef fishes in Okinawa Island, southern Japan. In: *University of Tokyo Bulletin, no. 25*. University of Tokyo Press, Tokyo, Japan. <http://umdb.um.u-tokyo.ac.jp/DKankoub/Bulletin/no25/no25000.html> (1984).

12. Tebbett SB, Goatley CHR, Bellwood DR. Clarifying functional roles: algal removal by the surgeonfishes *Ctenochaetus striatus* and *Acanthurus nigrofuscus*. *Coral Reefs* **36**, 803-813 (2017).

13. Carpenter KE, Niem VH. *The living marine resources of the Western Central Pacific. Volume 6: Bony fishes part 4 (Labridae to Latimeriidae)*. In: *FAO species identification guide for fishery purposes.* (FAO, Rome, Italy, 2001).

14. Marnane MJ, Bellwood D. Diet and nocturnal foraging in cardinalfishes (Apogonidae) at One Tree Reef, Great Barrier Reef, Australia. *Mar Ecol Prog Ser* **231**, 261-268 (2002).

15. Kulbicki M, Bozec YM, Labrosse P, Letourneur Y, Mou-Tham G, Wantiez L. Diet composition of carnivorous fishes from coral reef lagoons of New Caledonia. *Aquat Living Resour* **18**, 231–250 (2005).

16. McClanahan TR. Recovery of a coral reef keystone predator, *Balistapus undulatus*, in East African marine parks. *Biol Conserv* **94**, 191-198 (2000).

17. Young MAL, Bellwood DR. Fish predation on sea urchins on the Great Barrier Reef. *Coral Reefs* **31**, 731-738 (2012).

18. Fricke HW. Fische als feinde tropischer seeigel. *Mar Biol* **9**, 328-338 (1971).

19. Carpenter KE, Niem VH. *The living marine resources of the Western Central Pacific. Volume 5: Bony fishes part 3 (Menidae to Pomacentridae)*. In: *FAO species identification guide for fishery purposes.* (FAO, Rome, Italy, 2001).

20. Pratchett MS. Dietary overlap among coral-feeding butterflyfishes (Chaetodontidae) at Lizard Island, northern Great Barrier Reef. *Mar Biol* **148**, 373-382 (2005).

21. Berumen ML, Pratchett MS. Trade-offs associated with dietary specialization in corallivorous butterflyfishes (Chaetodontidae: *Chaetodon*). *Behav Ecol Sociobiol* **62**, 989-994 (2008).

22. Salini J, Blaber S, Brewer D. Diets of trawled predatory fish of the Gulf of Carpentaria, Australia, with particular reference to predation on prawns. *Mar Freshwater Res* **45**, 397-411 (1994).

23. Randall JE, Allen GR, Steene R. *Fishes of the Great Barrier Reef and Coral Sea*. 557 (Crawford House Press, Bathurst, Australia, 1997).

24. Froese R, Pauly D. *FishBase 2000: concepts, design and data sources*. 344 (Los Baños, Laguna, Philippines, 2000).

25. McClanahan TR. Fish predators and scavengers of the sea urchin *Echinometra mathaei* in Kenyan coral-reef marine parks. *Environ Biol Fishes* **43**, 187-193 (1995).

26. Randall JE, Head SM, Sanders APL. Food habits of the giant humphead wrasse, *Cheilinus undulatus* (Labridae). *Environ Biol Fishes* **3**, 235-238 (1978).

27. Layton C, Fulton CJ. Status-dependent foraging behaviour in coral reef wrasses. *Coral Reefs* **33**, 345-349 (2014).

28. Shibuno T, Hashimoto H, Gushima K. Changes with growth in feeding habits and gravel turning behavior of the wrasse, *Coris gaimard*. *Jap J Ichthyol* **41**, 301-306 (1994).

29. Connell SD. Patterns of pisciviory by resident predatory reef fish at One Tree Reef, Great Barrier Reef. *Mar Freshwater Res* **49**, 25-30 (1998).

30. Carpenter KE, Allen GR. *FAO Species Catalogue. Emperor fishes and large-eye breams of the world (family Lethrinidae). An annotated and illustrated catalogue of lethrinid species known to date.* FAO Fisheries Synopsis 125, Volume 9 (FAO, Rome, Italy, 1989).

31. Toor HS. Biology and fishery of the pig-face bream, *Lethrinus lentjan* (Lacépède). I. Food and feeding habits. *Indian J Fish* **11**, 559-580 (1964).

32. Walker MH. Food and feeding habits of *Lethrinus chrysostomus* Richardson (Pisces : Perciformes) and other Lethrinids on the Great Barrier Reef. *Mar Freshwater Res* **29**, 623-630 (1978).

33. Birdsey R. *Large reef fishes as potential predators of* Acanthaster planci*: A pilot study by alimentary tract analysis of predatory fishes from reefs subject to* Acanthaster *feeding*. 37 (Great Barrier Marine Park Authority, Townsville, Australia, 1988).

34. Brooker RM, Jones GP, Munday PL. Prey selectivity affects reproductive success of a corallivorous reef fish. *Oecologia* **172**, 409-416 (2013).

35. Boaden A, Kingsford M. Diel behaviour and trophic ecology of *Scolopsis bilineatus* (Nemipteridae). *Coral Reefs* **31**, 871-883 (2012).

36. Booth D, Alquezar R. Food supplementation increases larval growth, condition and survival of *Acanthochromis polyacanthus*. *J Fish Biol* **60**, 1126-1133 (2002).

37. Frédérich B, Liu S-YV, Dai C-F. Morphological and genetic divergences in a coral reef damselfish, *Pomacentrus coelestis*. *Evolutionary Biology* **39**, 359-370 (2012).

38. Ceccarelli DM, Emslie MJ, Lewis AR. Farming versatility by *Pomacentrus wardi*. *Mar Freshwater Res* **64**, 558–561 (2013).

39. Myers RF. *Micronesian reef fishes: a comprehensive guide to the coral reef fishes of Micronesia*. 3rd revised and expanded edition. 330 (Coral Graphics, Barragida, Guam, 1999).

40. Carpenter KE, Niem VH. *The living marine resources of the Western Central Pacific. Volume 4: Bony fishes part 2 (Mugilidae to Carangidae)*. In: *FAO species identification guide for fishery purposes.* (FAO, Rome, Italy, 2001).

41. St John J. Ontogenetic changes in the diet of the coral reef grouper *Plectropomus leopardus* (Serranidae): patterns in taxa, size and habitat of prey. *Marine Ecology-Progress Series* **180**, 233-246 (1999).

42. Hoey AS, Brandl SJ, Bellwood DR. Diet and cross-shelf distribution of rabbitfishes (f. Siganidae) on the northern Great Barrier Reef: implications for ecosystem function. *Coral Reefs* **32**, 973-984 (2013).

43. GBRMPA. Crown-of-thorns starfish control program. Great Barrier Reef Marine Park Authority, Townsville, Australia. <http://www.gbrmpa.gov.au/our-work/our-programs-and-projects/crown-of-thorns-starfish-control-program> (2019).

44. Rivera-Posada J, Pratchett MS, Aguilar C, Grand A, Caballes CF. Bile salts and the single-shot lethal injection method for killing crown-of-thorns sea stars (*Acanthaster planci*). *Ocean Coast Manage* **102**, 383-390 (2014).

45. Glynn PW. An amphinomid worm predator of the crown-of-thorns sea star and general predation on asteroids in eastern and western Pacific coral reefs. *Bull Mar Sci* **35**, 54–71 (1984).

46. Moran PJ. Preliminary observations of the decomposition of crown-of-thorns starfish, *Acanthaster planci* (L.). *Coral Reefs* **11**, 115-118 (1992).
